# Design and Synthesis of Novel Chalcone Derivatives Targeting Bladder and Liver Cancer: Cytotoxicity Evaluation and Preliminary Mechanistic Studies

**DOI:** 10.1021/acsomega.5c08480

**Published:** 2025-12-02

**Authors:** Aline Mol Hermenegildo, Izadora Amaral Nakao, Luana Beatriz Araújo Vaz, Gabrielly Guimarães Coutinho, João Lucas Moraes Toledo, Tamires Cunha Almeida, Tatiane Roquete Amparo, Kamila de Fatima Anunciação, Glenda Nicioli da Silva, Geraldo Célio Brandão, Saulo Fehelberg Pinto Braga, Thiago Belarmino de Souza

**Affiliations:** a School of Pharmacy, 28115 Federal University of Ouro Preto, Ouro Preto, MG 35402-163, Brazil; b Center for Development and Innovation, Butantan Institute, Sao Paulo, SP 05509-002, Brazil

## Abstract

The high incidence and mortality rates of bladder and liver cancers, combined with the challenges of early diagnosis, contribute to significant strain on healthcare systems. Standard chemotherapy treatments, which commonly use drug combinations, often cause intense side effects, reducing patient compliance and limiting therapeutic success. Considering the promising activity of a chalcone derivative of 2-methoxy-4-propylphenol against HepG2 cancer cells (hit compound **1**), discovered recently by our group, we synthesized new chalcone derivatives of this hit compound with three structural patterns to try to increase its activity, selectivity, and spectrum of action against other tumor lineages. Among the 12 new chalcones obtained, the *O*-methylated derivative **3** was the most promising one with IC_50_ values between 3.03 and 5.92 μM against all evaluated cancer cells (HeLa, HepG2, T24, and TOV-21G) and selective indices up to 18.2 considering the healthy MRC-5 cells studied. The cytotoxicity of **3** against these human cells was lower compared to the hit compound **1**, and when compared to the control drug doxorubicin, this new chalcone exhibited higher selectivity indices across all of the evaluated cells. In addition to cytotoxicity, the compound inhibited clonogenicity and migration in bladder and liver cancer cells, independent of metabolic capacity, while inducing cytostasis in the liver and early apoptosis in bladder cancer cells.

## Graphical abstract

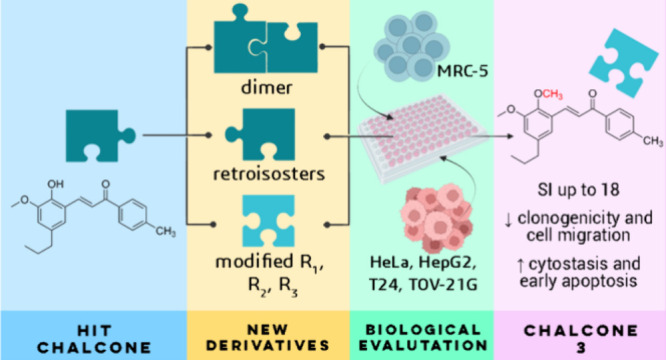

## 1 Introduction

Bladder and liver cancers rank as the 11th and 5th most prevalent malignancies worldwide and are the 14th and 4th leading causes of cancer-related deaths, respectively. Notably, the incidence of liver cancer has more than tripled since 1980.^1^

Bladder cancer develops through a multifactorial process involving a complex interaction between genetic predisposition and environmental influences. Among the various risk factors, smoking is the most significant as it contributes to the formation of DNA adducts that trigger genetic mutations. Other contributing factors include prolonged exposure to carcinogens, a family history of bladder cancer, chronic cystitis, and aging.^2^

Hepatocellular carcinoma (HCC), the most prevalent type of liver cancer, commonly develops in individuals with cirrhosis, chronic hepatitis B or C infections, long-term alcohol use, or nonalcoholic fatty liver disease. While the underlying causes may differ, the disease generally follows a similar progression: liver injury leads to chronic inflammation, followed by fibrosis, cirrhosis, and ultimately the development of HCC.^3^

The treatment of these tumors is often aggressive, with surgical intervention being a common approach. Nonetheless, surgery is associated with substantial risks such as elevated mortality and the possibility of metastatic spread. Moreover, the high toxicity and adverse side effects linked to conventional chemotherapy, along with significant recurrence rates, have driven the search for innovative therapeutic alternatives.^2,3^ Specifically, for bladder cancer, intravesical chemotherapy with mitomycin, immunotherapy, and systemic regimens such as gemcitabine plus cisplatin (GC) or methotrexate, vinblastine, doxorubicin, and cisplatin (MVAC) are standard treatments.^4^ For liver cancer, commonly used chemotherapy drugs include doxorubicin and cisplatin.^5^

Natural or synthetical chalcones, a class of α,β-unsaturated ketones, have attracted considerable attention in anticancer research^6–15^ due to their structural flexibility and diverse mechanisms of action, inducing cell cycle arrest at the G0/G1 phase, inhibiting the PI3K/Akt signaling pathway via suppression of Akt phosphorylation and upregulation of p21 and promoting apoptosis through NF-κB-p65 inhibition. Their structural adaptability also enables the design of analogues with enhanced pharmacokinetic profiles, reinforcing their suitability as candidates for new drug development.

There are other described mechanisms for anticancer activity of chalcones. The tumor suppressor protein p53 regulates the cell cycle and prevents cancer cell proliferation, and chalcones can upregulate p53 by disrupting p53-MDM2 interactions, modulating proteins like Sp1, CRM1, and HSP40, and restoring p53 pathways.^16,17^ Chalcones also act as antimitotic agents by binding to tubulin, inhibiting its polymerization and disrupting mitotic spindle assembly, leading to cell cycle arrest in the G2/M phase and apoptosis. Various chalcone derivatives, modeled as colchicine or combretastatin analogues, have shown potent antiproliferative and antivascular activity in vitro and in vivo, targeting the colchicine-binding site of β-tubulin.^18,19^ Some chalcones can inhibit angiogenesis, a key process in cancer progression, by modulating factors such as VEGF, bFGF, TGF-β, HIF-1, MMPs, and endothelial cell proliferation and migration.^20–22^ Other chalcones can overcome multidrug resistance (MDR) in cancer by inhibiting key ATP-binding cassette (ABC) transporters, including P-glycoprotein (P-gp/ABCB1), MRP1 (ABCC1), and BCRP (ABCG2).^23–26^

Recently, our research group discovered a chalcone derived from 2-methoxy-4-propylphenol (**1**; Figure 1) active against HepG2 (human liver hepatocellular carcinoma; IC_50_: 4.2 μM). This compound showed a selectivity index greater than 11 for HepG2 cells considering the healthy MRC-5 (human lung fibroblast) cells evaluated. Its abilities of inducing cell death and inhibiting HepG2 cell migration were also confirmed, and in silico studies suggested the metalloproteinase MMP-9 as the molecular target for this antimigratory potential.^27^ Considering this promising potential of chalcone **1**, we designed new chalcone derivatives of this hit compound with three structural patterns shown in Figure 1: modifications in R_1_, R_2_, and R_3_ positions, a dimer, and retroisosteric derivatives, in an attempt to find new compounds that are more potent and selective for cancer cells.

**Figure 1:**
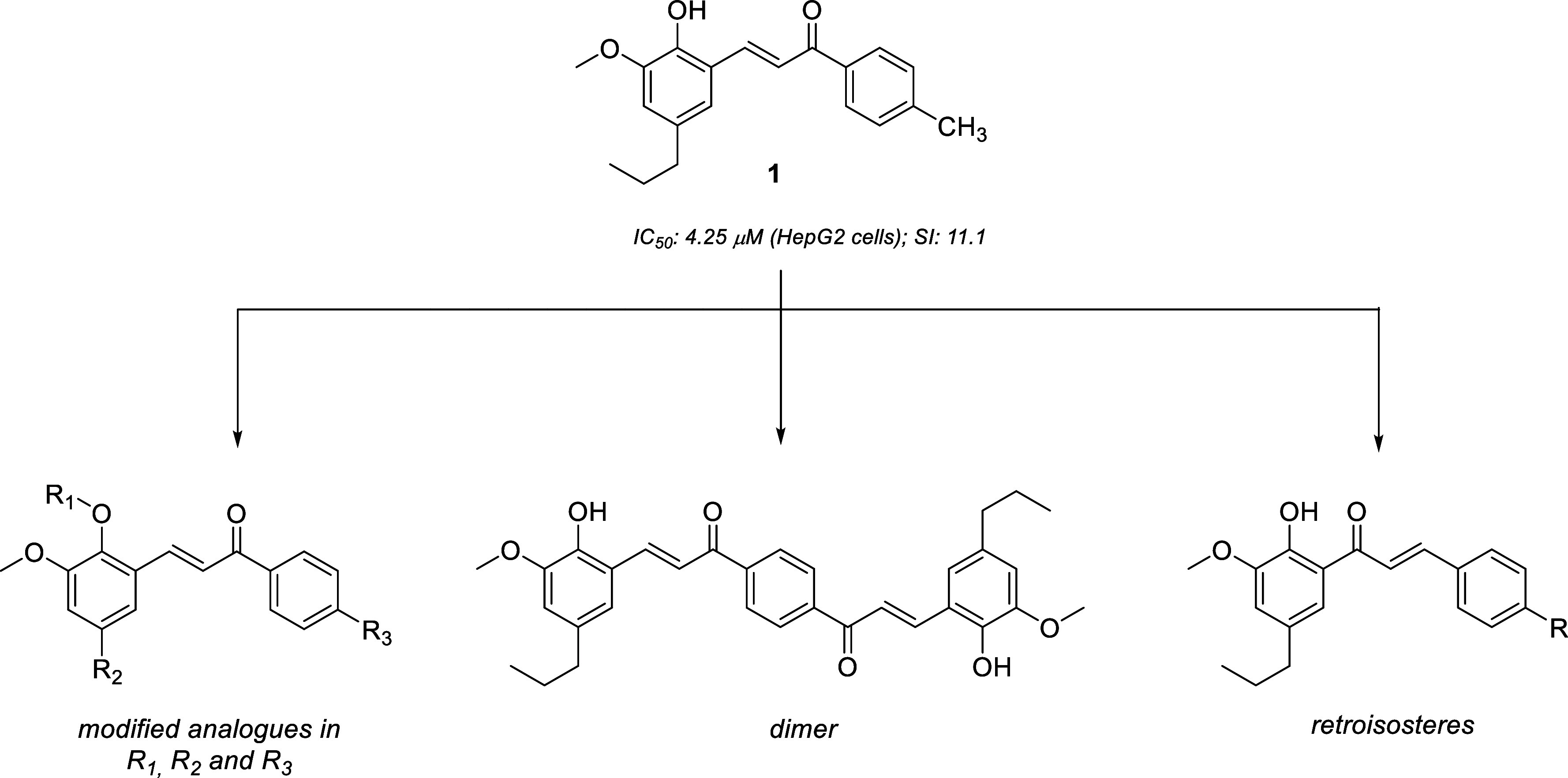
Bioactive hit chalcone (**1**) and newly designed derivatives.

## 2 Results and Discussion

### 2.1 Chemistry

The new chalcones were synthesized according to the synthetic routes shown in Schemes 1–4 below. Initially, to obtain the new chalcones modified in the R_1_ position (Figure 1), the hit chalcone **1** was converted to three *O*-substituted derivatives: the acetylated derivative **2** was obtained from the reaction of **1** with acetic anhydride,^28^ while the alkylated derivatives **3** and **4** were obtained after the reaction of **1** with iodomethane or benzyl chloride, respectively.^29^ The synthetic route and yields for obtaining these compounds are shown in Scheme 1.

**Scheme 1:**
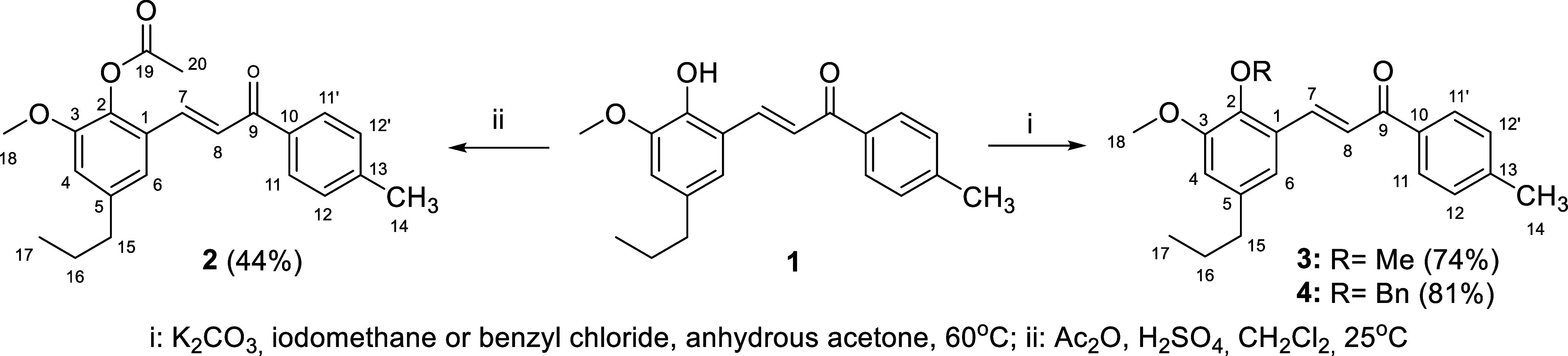
Synthesis of *O*-Substituted Chalcones **2**–**4**

**Scheme 2:**
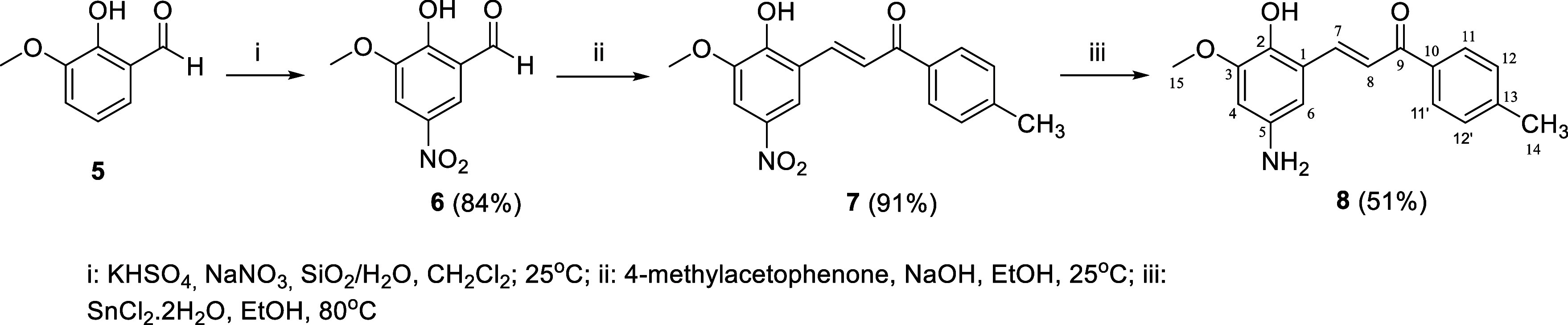
Synthesis of Nitro (**7**) and Amine (**8**) Chalcones

**Scheme 3:**
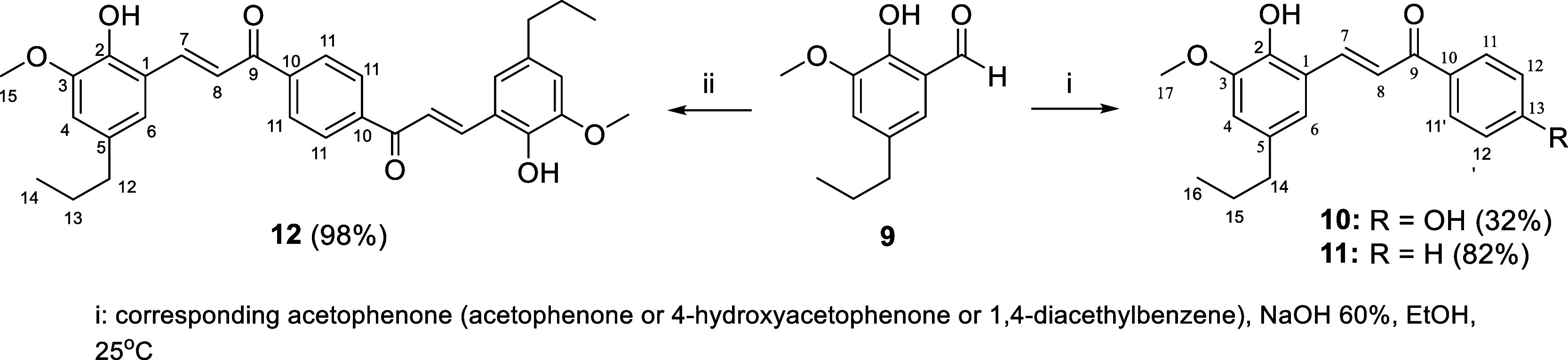
Synthesis of R_3_-Modified (**10** and **11**) and Dimer (**12**) Chalcones

**Scheme 4:**
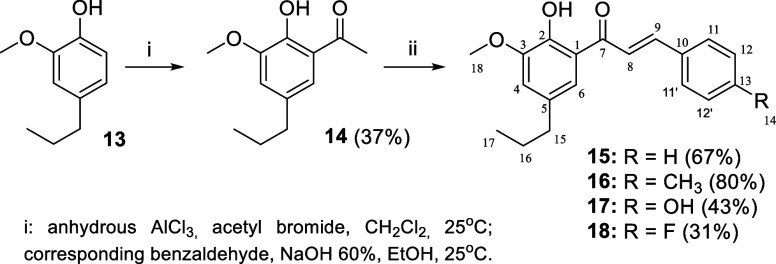
Synthesis of Retroisosteric Chalcones **15**–**18**

The chalcones modified at the R_2_ position (Figure 1) were obtained using the route presented in Scheme 2, in which *ortho*-vanillin (**5**) was subjected to a nitration reaction with sodium nitrate and potassium bisulfate.^30^ The reaction of the obtained nitro derivative (**6**) with 4-methylacetophenone afforded the chalcone **7**,^31^ which after a nitro reduction condition in the presence of tin chloride^32^ led to the production of amino chalcone **8**.

The R_3_-modified and dimer chalcones (Figure 1) were obtained as shown in Scheme 3. While the derivatives **10** and **11** were synthesized by reaction of formyl-dihydroeugenol **9** as previously described^32^ with acetophenone or 4-hydroxyacetophenone, in the presence of sodium hydroxy, the dimer **12** was obtained using 1,4-diacetylbenzene and an excess of **9**.^32^

Finally, the retroisosteric chalcones (Figure 1) were synthesized from two steps. For this, first, the dihydroeugenol (**13**) was *C*-acylated using acetyl bromide^33^ and the reaction of the obtained acylated derivative **14** with different *para*-substituted benzaldehydes^32^ afforded the retroisosteric chalcones **15**–**18**, as shown in Scheme 4.

In infrared spectra of new chalcones, it was possible to observe a band near 1660 cm^–1^ corresponding to the carbonyl groups of these compounds. In the ^1^H NMR spectra, two doublets with a coupling constant near 16 Hz were registered for all of the new compounds, referring to the *trans*-olefin system. Th signals referring to the carbonyl group were observed between 187 and 196 ppm in the ^13^C NMR spectra. All of the other signals were properly identified for the compounds, in both the ^1^H and ^13^C NMR spectra.

### 2.2 Cytotoxicity Studies and Structure–Activity Relationships

The global burden of cancer continues to rise, with liver, bladder, ovarian, and cervical cancers representing major public health challenges. The selection of cancer cell lines in the present study was based on their epidemiological relevance, clinical severity, and availability of well-established in vitro models.

According to the World Health Organization, liver cancer was one of the most common malignancies in men in 2018 and, by 2020, ranked among the leading causes of cancer-related deaths worldwide, accounting for approximately 830,000 deaths. It remains the sixth most frequently diagnosed cancer overall and the primary cause of cancer mortality when both sexes are considered.^34^ Bladder cancer was the 10th most prevalent malignancy in 2018 and, in 2023, was estimated to account for 82,290 new cases (4.2% of all new diagnoses) and 16,710 deaths.^35^ Ovarian cancer is also of significant concern, with 19,710 new cases and 13,270 deaths projected for 2023. Cervical cancer, largely linked to persistent human papillomavirus (HPV) infection, ranks as the fourth most frequently diagnosed cancer in women worldwide, with more than 600,000 new cases and approximately 350,000 deaths in 2022, disproportionately affecting low- and middle-income countries.^35^

To evaluate the cytotoxicity of the synthesized compounds, the nontumoral human lung fibroblast line MRC-5 was included as a control. MRC-5 cells are widely used in biomedical research due to their robust in vitro growth, cytogenetic stability, and nontumorigenic profile.^36,37^ Importantly, fibroblasts are highly sensitive to antineoplastic chemotherapies, providing a relevant model for assessing compound selectivity.^38^

Cytotoxicity was determined using the MTT colorimetric assay, and half-maximal inhibitory concentrations (IC_50_, μM) were obtained for all cell lines (Table 1). The selectivity index (SI) was calculated as the ratio of IC_50_ MRC-5/IC_50_ cancer cell line. Doxorubicin was employed as a positive control due to its broad-spectrum clinical use, either alone or in combination, against hepatocellular, ovarian, cervical, and bladder cancers.^39–41^

**Table 1:** Cytotoxic Activities of Obtained Chalcones against MRC-5, HeLa, HepG2, T24, and TOV-21G Cells (IC_50_), Standard Deviations (*n* = 3), and Selectivity Indices (SI)^a^

	IC_50_ μM								
compound	MRC-5	HeLa	SI	HepG2	SI	T24	SI	TOV-21G	SI
**1** (hit)	47.2 ± 1.3	NT	—	4.2 ± 1.3	11.1	>161 ± 1.47	<0.2	7.2 ± 1.3	6.5
**2**	16.1 ± 1.5	3.6 ± 1.3	4.7	4.2 ± 1.2	3.8	59.5 ± 1.5	0.2	1.2 ± 1.1	13.3
**3**	55.5 ± 1.2	5.9 ± 1.6	9.3	4.5 ± 1.2	12.2	5.0 ± 1.6	11.1	3.0 ± 2.6	18.2
**4**	242.4 ± 2.4	NA	—	16.9 ± 1.3	14.3	NA	—	NA	—
**7**	28.1 ± 1.3	26.6 ± 1.8	1.0	16.6 ± 1.5	1.6	103.8 ± 1.9	0.2	23.8 ± 2.8	1.1
**8**	40.9 ± 2.0	11.5 ± 1.8	3.5	NA	—	158.6 ± 1.4	0.2	53.1 ± 2.6	0.7
**10**	5.0 ± 1.3	17.9 ± 2.0	0.2	18.9 ± 1.6	0.2	34.9 ± 1.4	0.1	7.2 ± 1.2	0.7
**11**	11.0 ± 1.2	2.1 ± 1.1	5.2	4.6 ± 1.2	2.3	9.2 ± 1.2	1.2	11.9 ± 1.5	0.9
**12**	35.0 ± 1.5	20.4 ± 1.5	1.7	NA	—	10.4 ± 1.7	3.3	33.7 ± 2.4	1.0
**15**	18.4 ± 1.9	13.7 ± 1.5	1.3	16.1 ± 1.2	1.1	18.4 ± 1.9	1	5.4 ± 1.2	3.3
**16**	7.7 ± 1.5	12.8 ± 1.3	0.6	6.6 ± 1.4	1.1	17.1 ± 2.1	0.4	10.9 ± 1.5	0.7
**17**	12.7 ± 1.4	114.2 ± 2.1	0.1	19.4 ± 1.4	0.6	36.4 ± 1.5	0.3	39.0 ± 1.5	0.3
**18**	11.5 ± 1.2	4.1 ± 1.3	2.8	8.3 ± 1.2	1.3	15.4 ± 1.5	0.7	4.8 ± 1.4	2.3
doxorubicin	0.6 ± 1.2	2.9 ± 1.1	0.2	0.06 ± 1.2	1.0	4.4 ± 1.3	0.1	0.5 ± 1.6	1.3

a Compound **1**: hit chalcone; IC_50_: 50% cytotoxic concentration; NT: no test compounds; NA: noncytotoxic at the concentrations tested; MRC-5: human lung fibroblast cells; HepG2: human liver hepatocellular carcinoma cells; HeLa: human cervical adenocarcinoma cells; T24: human urinary bladder carcinoma cells; TOV-21G: human ovarian adenocarcinoma cells.

Considering the results presented in Table 1, it is evident that replacing the phenolic hydroxyl group of chalcone hit **1** with smaller lipophilic groups, such as acetyl (compound **2**) or methyl (compound **3**), significantly enhanced its cytotoxic activity against the T24 and TOV-21G cell lines. The *O*-acetylated derivative **2** was over six times more active against TOV-21G cells (IC_50_ of 7.2 μM for **1** vs 1.2 μM for **2**), while the *O*-methylated derivative **3** demonstrated more than 32 times greater potency against T24 cells (IC_50_ > 161 μM for **1** vs 5.0 μM for **3**). However, this increase in cytotoxicity was not observed when the phenolic hydroxyl was replaced by a bulkier group, as evidenced by the low activity of benzylated derivative **4**.

Interestingly, derivative **3** showed activity against all four cancer cell lines tested, with effects near the IC_50_ values, including against HeLa cells, which were not assessed for the hit compound in our previous study.^6^ The cytotoxicity of derivative **3** against the evaluated human MCR-5 cells was lower compared to that of the hit compound **1**, which resulted in higher selectivity index values for this *O*-methylated derivative (ranging from 9.3 to 18.2) considering all the cancer cell lines tested. Finally, compared to the control drug doxorubicin, derivative **3** exhibited higher selectivity indices across all evaluated cancer cells, indicating lower toxicity and enhanced safety relative to that of this drug.

Replacing the propyl group in chalcone **1** with a nitro (compound **7**) or amino (compound **8**) group significantly reduced their cytotoxic activity against cancer cells and increased toxicity toward healthy MRC-5 cells, highlighting the critical role of the propyl group in maintaining both activity and selectivity. Another substitution showing a similar pattern was replacing the *para*-methyl group of chalcone **1** with a hydroxyl group (compound **10**) or removing it altogether (compound **11**), both of which resulted in reduced activity and increased toxicity of these compounds.

Dimerization of chalcone **1** (compound **12**) resulted in increased cytotoxic activity only against T24 cells (IC_50_ > 161 μM for **1** vs 10.4 μM for **12**), and the synthesis of retroisosteric chalcones (compounds **15**–**18**) did not significantly enhance the cytotoxic activity of these compounds against cancer cells, but it did increase their toxicity toward healthy MRC-5 cells.

Natural and synthetic chalcone derivatives have been extensively reported to exert potent anticarcinogenic activity across multiple tumor models. Previous studies have demonstrated that chalcones induce cytotoxicity and effectively inhibit proliferation and migration in hepatocellular^42^ and bladder carcinoma cells,^43^ in addition to showing marked cytotoxic effects against cervical (HeLa)^44^ and ovarian cancer cells.^45^ These findings reinforce the relevance of this chemical class as a promising scaffold for anticancer drug development.

In the present study, the *O*-methylated derivative **3** displayed a broad spectrum of activity, accompanied by selectivity indices, when compared with the other tested compounds. Such characteristics highlight its potential therapeutic value and justify its prioritization for more comprehensive biological characterization. Accordingly, derivative **3** was selected for subsequent functional assays, including clonogenic survival, cell migration, assessment of morphological alterations, analysis of cell cycle progression, and apoptosis/necrosis induction. These complementary studies are essential to elucidate the cellular mechanisms underlying its cytotoxic profile and to provide insights into its potential applicability as an antitumor candidate.

### 2.3 Clonogenic Survival

The results showed a reduction in colony formation in HepG2 cells at concentrations of 4.62 and 9.25 μM. For the T24 cells, a significant effect was observed at the maximum concentration tested (18.50 μM) (Figure 2).

**Figure 2:**
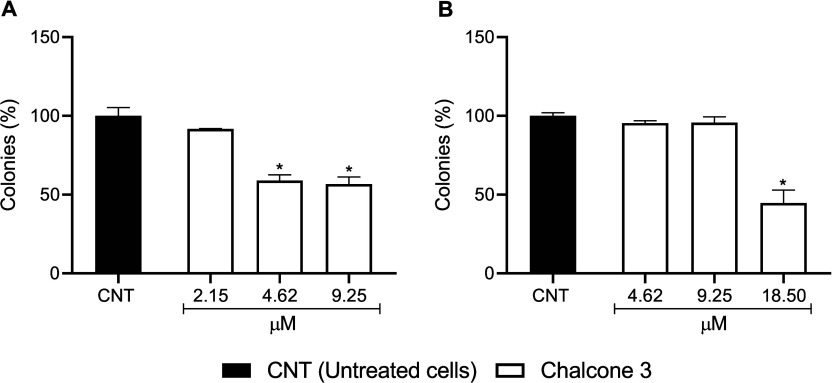
Effect of chalcone **3** on the clonogenic viability of the HepG2 (A) and T24 (B) cell lines. *p < 0.05 compared to the untreated cells (CNT) determined by one-way ANOVA followed by Dunnett’s post-test. Experiments were carried out in triplicate, and the results are expressed as means and SD (bars).

Several authors have also reported the antiproliferative and anticancer properties of natural chalcones across various malignancies, including breast cancer, gastrointestinal cancers, lung cancer, renal and bladder cancers, and melanoma.^46^

### 2.4 Cell Migration

The results showed reduction of cell migration at concentrations of 4.62 and 9.25 μM for HepG2, with a more pronounced effect at the maximum concentration. For T24 cells, a significant decrease was observed at the highest concentration tested (Figure 3).

**Figure 3:**
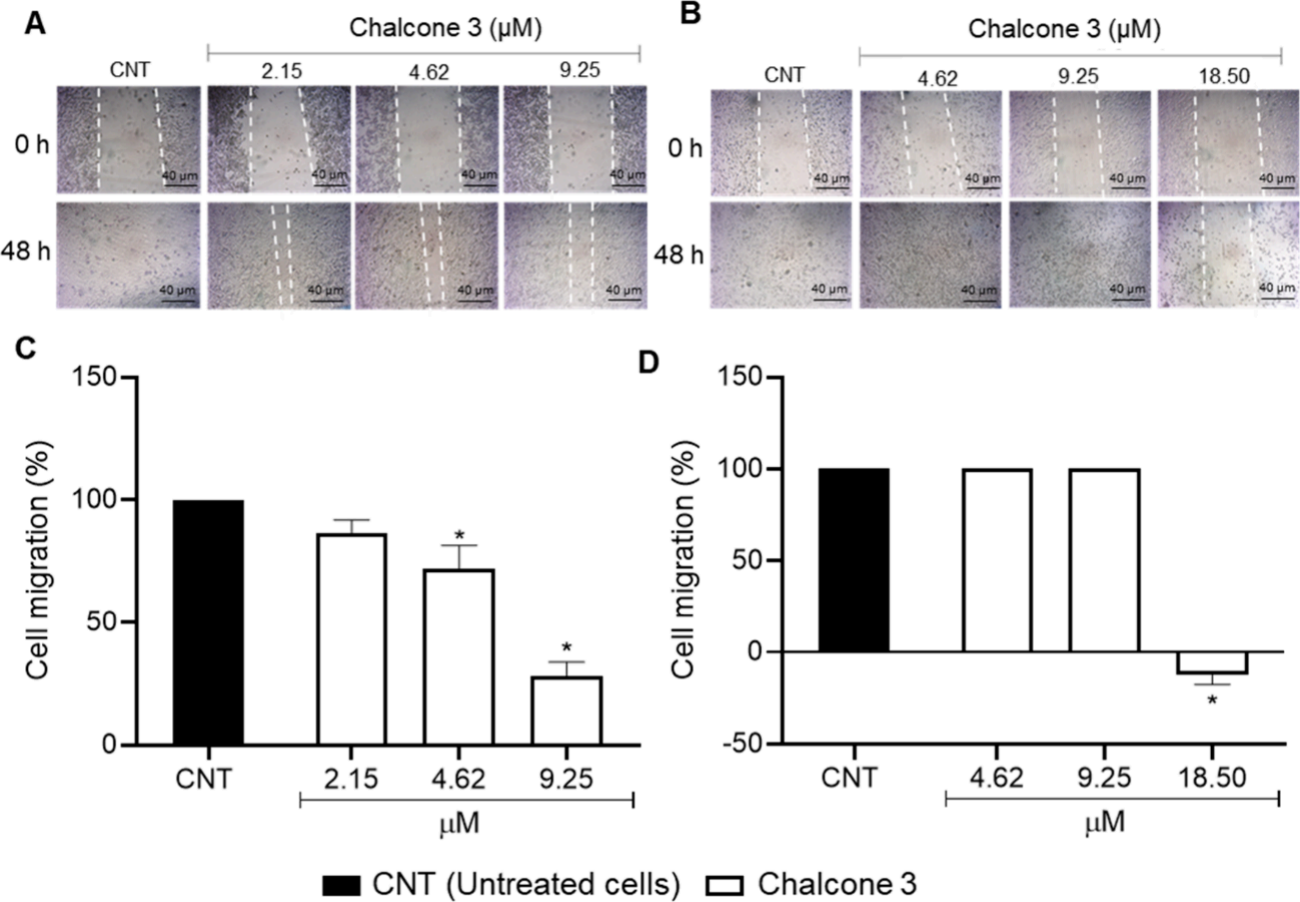
Effect of chalcone **3** on the migration of HepG2 (A, C) and T24 (B, D) cell lines. *p < 0.05 compared to the untreated cells (CNT) determined by one-way ANOVA followed by Dunnett’s post-test. Experiments were carried out in triplicate, and the results are expressed as means and SD (bars).

These observations indicate context-dependent cellular responses rather than broad selectivity and are consistent with previous studies reporting variability in chalcone sensitivity among tumor cell types. For instance, Li and co-workers demonstrated differences in the sensitivity of various tumor cell lines to modified chalcones, with hepatocarcinoma cells showing greater susceptibility at suboptimal concentrations than other tumor types.^47^ Likewise, Tsai and colleagues reported that licochalcone A (LCA), at subtoxic concentrations (5–20 μM), effectively inhibited migration and invasion of human hepatocellular carcinoma (HCC) cells, associated with decreased uPA protein and mRNA expression.^48^ Considering uPA’s role in extracellular matrix degradation, such findings support the notion that certain chalcones may exert antimigratory or antimetastatic effects, although the underlying mechanisms remain to be fully elucidated.

### 2.5 Computational Studies

Target prediction using SwissTargetPrediction yields only low-probability candidates.^49^ Thus, based on the structural similarity to the hit chalcone, we hypothesized that the two neighboring oxygen atoms on the aromatic ring might coordinate the catalytic zinc ion of metalloproteinases, thereby contributing to its antimigratory effect. As with the hit chalcone, we also performed 25 ns molecular dynamics (MD) simulations of chalcone **3** on an MMP-9.

The reference structure (PDB 4xct) cocrystallized with an inhibitor shows stable hydroxamate-Zn^2^^+^ coordination, with mean Zn–O distances of 2.17 and 2.52 Å.^50^ In contrast, the chalcone **3** complex was unstable, displaying a mean RMSD of 5.31 Å from the docking pose (Figure 4A). Additionally, no coordination with the metal was observed, with average Zn–O distances of 8.68 and 10.54 Å (Figure 4B). Trajectory analysis indicates that the methoxylated ring is expelled from the binding site after 5 ns. Compared with the parent hit chalcone **1**, substitution of the hydroxy group by methoxy appears to introduce steric hindrance that impairs zinc coordination.^27^

**Figure 4:**
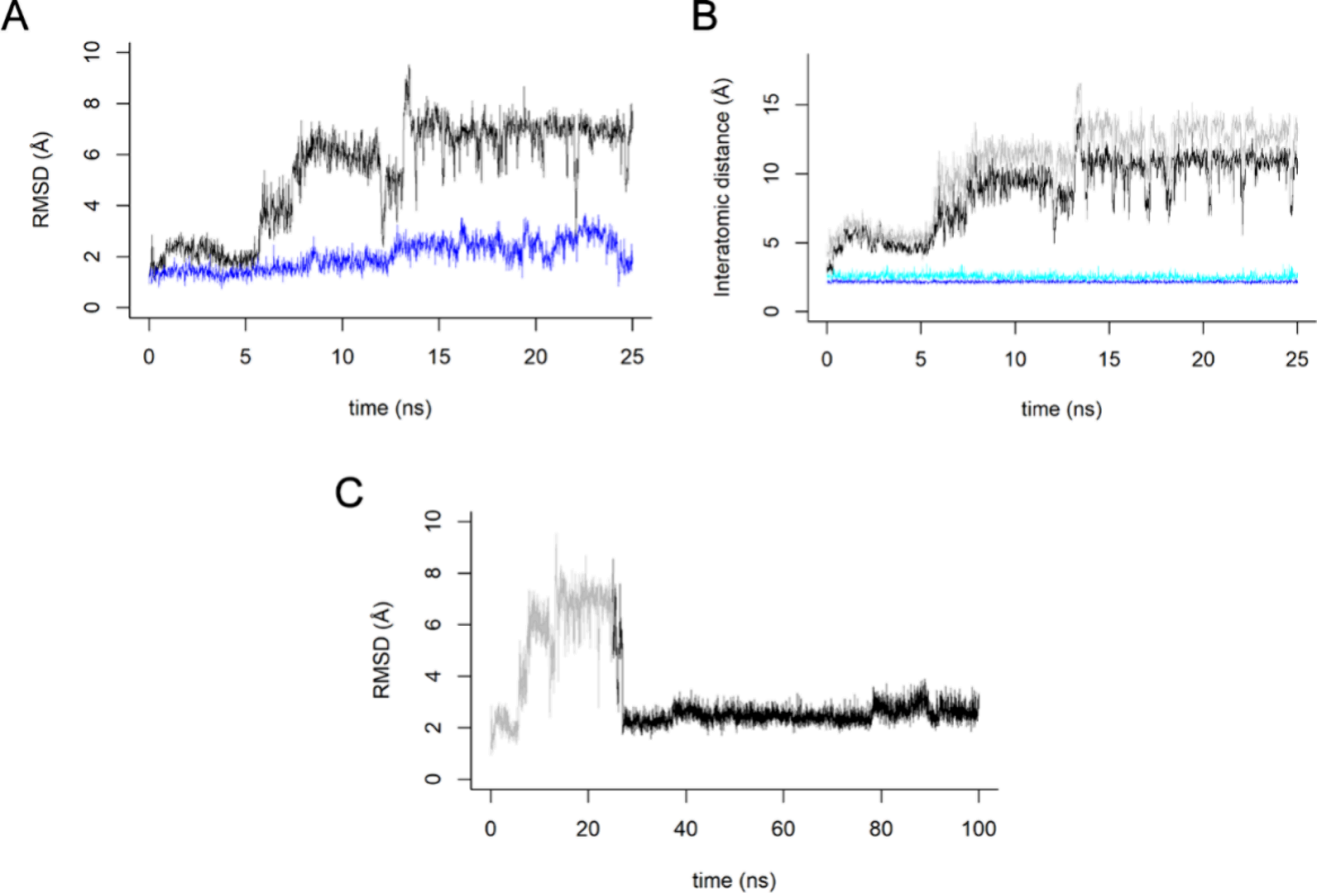
Molecular dynamics (MD) analysis. RMSD of chalcone **3** (black) and MMP-9 inhibitor (blue) nonhydrogen atoms from their docked/crystallographic poses over 25 ns (A). Interatomic distances between the catalytic Zn^2^^+^ ion and the methoxyl oxygens of chalcone **3** (black and gray) and the hydroxamate oxygens of the MMP-9 inhibitor (blue and cyan) over 25 ns (B). RMSD of chalcone **3** over the 100 ns extended simulation (black) compared with the initial 0–25 ns trajectory (gray) (C).

Nevertheless, the tolyl group remains stabilized by hydrophobic residues (Leu22, Val223, Val243, and Tyr248), which prevents complete dissociation from the binding pocket. Notably, in a 100 ns extended simulation, the ligand reoriented back toward its original position, with the RMSD falling below 3 Å at the end of the run (Figure 4C). These results suggest that chalcone **3** might have a low affinity to inhibit MMP-9 directly via zinc coordination, and further studies are needed to explain the molecular basis of its antimigratory activity.

Furthermore, preliminary pharmacokinetic evaluation was carried out on the SwissADME platform.^51^ Although chalcone **3** passed Lipinski and Veber rules, its high lipophilicity (consensus log *P* of 4.68) and moderate to poor water solubility prediction raise concerns, suggesting that more polar derivatives should be considered in future optimizations (Table S1). While the small hydrophobic methoxy substituent may contribute to cytotoxic effects, polar functional groups such as hydroxamate could enhance zinc affinity and increase polarity, providing a more favorable pharmacokinetic profile.^52^

### 2.6 Morphology

The morphological assay enables the visualization and analysis of treatment-induced alterations in the cellular morphology. In both cell lines, a reduction in cell density was observed following treatment, aligned with the findings of the cytotoxicity assay. Furthermore, the presence of cell debris, rounded cells, and elongated cells, features commonly associated with cell death, was noted (Figure 5).

**Figure 5:**
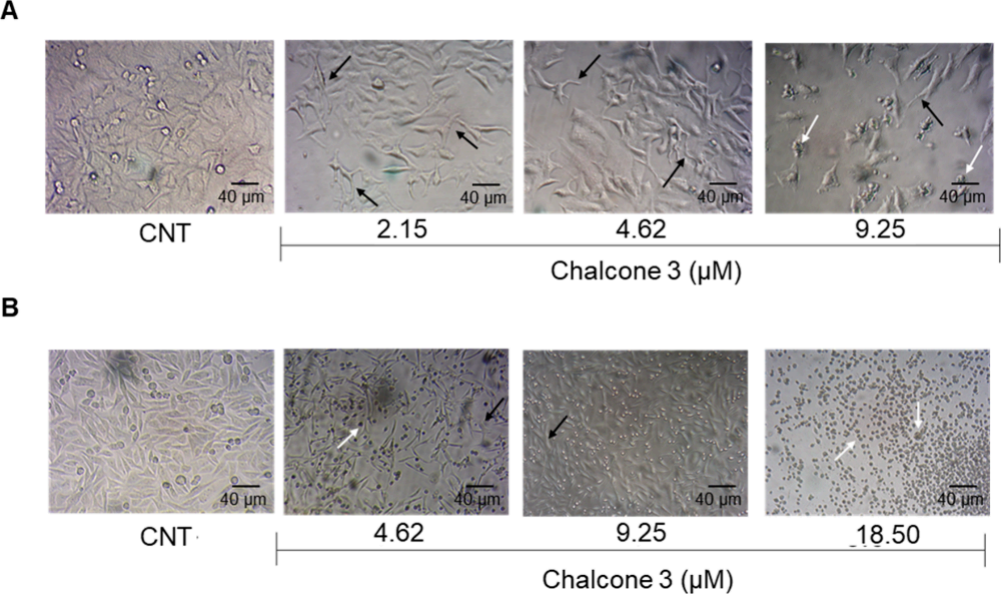
Morphological changes in HepG2 (A) and T24 (B) cell lines after treatment with chalcone **3**. White arrows are cell debris, indicating cell death; black arrows are elongated cells, suggesting changes in the cytoskeleton.

### 2.7 Cell Cycle Progression

In the HepG2 cells, a significant increase in the population of cells in the G0/G1 phase was observed at all concentrations tested (0.92–4.62 μM), accompanied by a corresponding reduction in the G2/M phase. This pattern suggests that the compound induces a block at the G1/S checkpoint, characteristic of agents that interfere with the cell cycle progression. Notably, the fraction of cells in sub-G1 (indicative of apoptosis) remained stable, indicating that the primary effect in this cell line is cytostatic at the concentrations evaluated. In contrast, the T24 cells showed a distinct response profile; no difference was observed after treatment (Figure 6).

**Figure 6:**
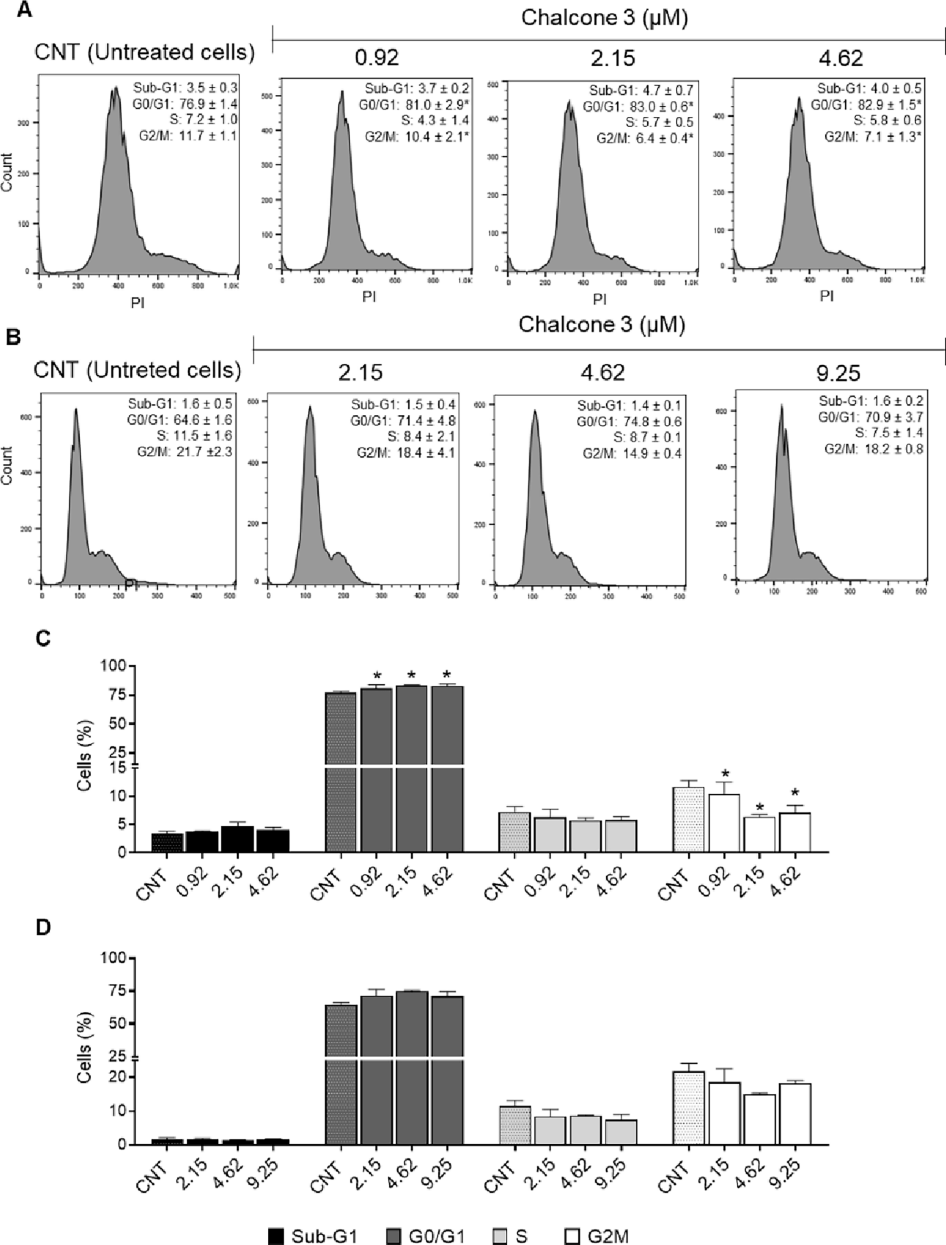
Analysis of the effect of chalcone **3** on the cell cycle distribution of HepG2 (A, C) and T24 (B, D) cells. *p < 0.05 compared to the untreated cells (CNT) determined by one-way ANOVA followed by Dunnett’s post-test. Experiments were carried out in triplicate, and the results are expressed as means and SD (bars).

The results revealed differential sensitivity between the two cell lines to chalcone **3**. HepG2 cells exhibited a more pronounced response, with G1 phase arrest, while T24 cells were relatively more resistant, likely due to intrinsic differences in cell cycle control or detoxification mechanisms. These findings suggest context-dependent cellular responses^53^ rather than broad selectivity and point to potential avenues for further mechanistic exploration.

### 2.8 Apoptosis/Necrosis Analysis

Consistent with the observations regarding cell cycle progression, the modality of cell death induced by chalcone **3** was cell line-dependent. In HepG2 cells, treatment with compound **3** at concentrations of 2.15, 4.62, or 9.25 μM did not result in a significant increase in the percentage of apoptotic or necrotic cells (Figure 7A). These findings suggest that other mechanisms of cell death, not detectable by annexin V and propidium iodide staining, may be involved in the effects of **3**, at the tested concentrations, on this cell line. For example, another chalcone derivative caused massive autophagy in HepG2 cells^54^ while other chalcones were able to induce cell death through ferroptosis in different tumor types.^55–57^

**Figure 7:**
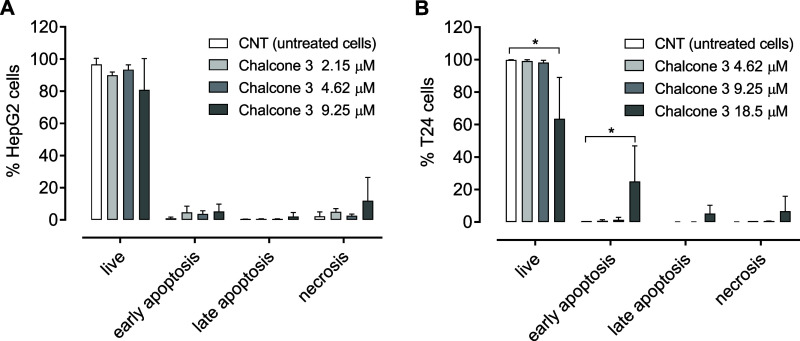
Percentage of viable, apoptotic, and necrotic HepG2 (A) and T24 (B) cells after the treatment with chalcone **3** for 48 h. *p < 0.05 compared to the untreated cells (CNT) determined by one-way ANOVA followed by Dunnett’s post-test. Experiments were carried out in triplicate, and the results are expressed as means and SD (bars).

On the other hand, in T24 cells, chalcone **3** at 18.50 μM reduced cell viability and increased the proportion of cells undergoing early apoptosis (Figure 7B). Interestingly, this effect is likely mediated through p53-independent pathways since T24 cells harbor a TP53 mutation resulting in an in-frame deletion of tyrosine 126.^58^

## 3 Conclusions

Twelve novel chalcones were synthesized based on the structure of the bioactive prototype chalcone **1**. Among the compounds obtained, the *O*-methylated derivative **3** exhibited cytotoxic activity stronger than that of the prototype against four cancer cell lines, along with higher selectivity indices. Beyond its cytotoxic effects, compound **3** inhibited clonogenicity and migration in both bladder and hepatic cancer cells, independent of metabolic capacity, and additionally induced cytostatic modulation in hepatic cancer cells and early apoptosis in bladder cancer cells. However, the molecular targets underlying these biological effects have not yet been elucidated, and further studies are warranted to clarify the mechanisms of action of these chalcones.

## 4 Materials and Methods

### 4.1 General Procedures

Melting points of chalcones and isoxazoles were determined on a Reichert Austria apparatus and were uncorrected. IR spectroscopy was performed on a Shimadzu FTIR-Affinity-1 spectrometer. ^1^H NMR and ^13^C NMR spectra were obtained on a Bruker AC-400 spectrometer (400 MHz for ^1^H NMR and 100 MHz for ^13^C spectra) in deuterated chloroform. Chemical shifts (δ) were reported in parts per million (ppm) with reference to tetramethylsilane (TMS) as internal standard, and coupling constants (*J*) were reported in Hertz (Hz). The following abbreviations were used for the ^1^H multiplicities: singlet (s), doublet (d), double doublet (dd), triplet (t), sextet (sext), and multiplet (m). High-resolution mass spectra (HRMS) were acquired using an LCMS-Q-ORBITRAP mass spectrometer, and the samples were solubilized in MeOH 50%, following manual injection. Reaction courses and product mixtures were monitored by thin-layer chromatography (TLC) on commercial silica gel 60 plates. For chromatography, column-grade silica gel (0.040–0.063 mm mesh size) was employed.

### 4.2 Synthesis of the Compounds

#### 4.2.1 Synthesis of Compound **2**

To 1 equiv (100 mg, 0.322 mmol) of chalcone **1** solubilized in 10 mL of dichloromethane was added 10 equiv (328 mg, 3.22 mmol) of acetic anhydride and H_2_SO_4_ (3 drops), and the reaction was stirred for 24 h at 25 °C. After this time, it was possible to observe the total consumption of the starting material. The mixture was washed with an aqueous solution of 10% NaOH (3 × 20 mL) and water (2 × 50 mL). The resulting organic phase was dried by anhydrous sodium sulfate and concentrated in a rotary evaporator.

##### 4.2.1.1 (*E*)-2-Methoxy-6-(3-oxo-3-(*p*-toluyl)­prop-1-en-1-yl)-4-propylphenyl Acetate (**2**)

This product was obtained in 44% yield as a green solid after purification by recrystallization in ethyl alcohol. IR-ATR (cm^–1^): 2962, 2920, 2871 (C–H sp^3^), 1758 (C=O ester), 1658 (C=O ketone), 1598, 1585 (Ar. C=C), 1282 (Ar–O–CH_3_), 1180 (C–O). ^1^H NMR (CDCl_3_, 400 MHz): δ 7.93 (d, 2H, ^3^ *J* = 8.12 Hz, H-11, H-11′), 7.83 (d, 1H, ^3^ *J* = 15.76 Hz, H-7), 7.50 (d, 1H, ^3^ *J* = 15.76 Hz, H-8), 7.31 (d, 2H, ^3^ *J* = 7.96 Hz, H-12, H-12′), 7.14 (br s, 1H, H-4), 6.82 (d, 1H, ^4^ *J* = 1.2 Hz, H-6), 3.83 (s, 3H, H-18), 2.60 (t, 2H, ^3^ *J* = 7.52 Hz, H-15), 2.43 (s, 3H, H-14), 2.37 (s, 3H, H-20), 1.68 (sext, 2H, ^3^ *J* = 7.36 Hz, H-16), 0.98 (t, 3H, ^3^ *J* = 7.32 Hz, H-17). ^13^C NMR (CDCl_3_, 100 MHz): δ 189.8 (1C, C-9), 168.8 (1C, C-19), 151.2 (1C, C-3), 143.7 (1C, C-13), 141.3 (1C, C-7), 137.8 (1C, C-2), 137.2 (1C, C-5), 135.4 (1C, C-10), 129.3 (1C, C-8), 128.6 (2C, C-11, C-11′), 128.3 (2C, C-12, C-12′), 124.0 (1C, C-1), 118.4 (1C, C-6), 114.1 (1C, C-4), 56.0 (1C, C-18), 38.1 (1C, C-15), 24.5 (1C, C-16), 21.6 (1C, C-14), 20.5 (1C, C-20), 13.8 (1C, C-17). HRMS-ESI: *m*/*z* calcd. for C_22_H_24_O_4_ (M + H)^+^, 353.1798; found, 353.1743.

#### 4.2.2 General Procedure for Synthesis of Compounds **3** and **4**

To 1 equiv (100 mg, 0.322 mmol) of chalcone **1** solubilized in 15 mL of anhydrous acetone was added 3 equiv (133 mg, 0.966 mmol) of potassium carbonate, and the reaction mixture was stirred for 30 min at 25 °C. After this time, 3 equiv (60 μL, 0.966 mmol) of methyl iodide (for synthesis of **3**) or 5 equiv (185 μL, 1.61 mmol) of benzyl chloride (for synthesis of **4**) was added to the mixture dropwise. The reaction was retained under magnetic stirring for 24 h until the total consumption of the starting chalcone was achieved. The resulting solid (K_2_CO_3_) was removed by filtration, and the filtrate was concentrated, resolubilized in 30 mL of dichloromethane, and washed with distilled water (2 × 30 mL). The resulting organic phase was dried over anhydrous sodium sulfate and concentrated on a rotary evaporator.

##### 4.2.2.1 (*E*)*-*3-(2,3-Dimethoxy-5-propylphenyl)-1-(*p*-tolyl)­prop-2-en-1-one (**3**)

This product was obtained in 74% yield as a white solid after purification by column chromatography (ethyl acetate/methanol 99.5:0.5 v/v). IR-ATR (cm^–1^): 2958, 2929, 2869 (sp^3^ C–H), 1660 (ketone C=O), 1593,1481 (Ar. C=C), 1280 (sp^3^ C–H). ^1^H NMR (CDCl_3_, 400 MHz): δ 8.06 (d, 1H, ^3^ *J* = 15.88 Hz, H-7), 7.95 (d, 2H, ^3^ *J* = 8.16 Hz, H-11, H-11′), 7.58 (d, 1H, ^3^ *J* = 15.88 Hz, H-8), 7.30 (d, 2H, ^3^ *J* = 7.96 Hz, H-12, H-12′), 7.07 (d, 1H, ^4^ *J* = 1.52 Hz, H-3), 6.78 (d, 1H, ^4^ *J* = 1.72 Hz, H-5), 3.88 (s, 3H, H-19), 3.85 (s, 3H, H-18), 2.58 (t, 2H, ^3^ *J* = 6 Hz, H-15), 2.43 (s, 3H, H-14), 1.66 (sext, 2H, ^3^ *J* = 7.44 Hz, H-16), 0.97 (t, 3H, ^3^ *J* = 7.32 Hz, H-17). ^13^C NMR (CDCl_3_, 100 MHz): δ 190.4 (1C, C-9), 152.8 (1C, C-2), 146.9 (1C, C-1), 143.4 (1C, C-13), 139.8 (1C, C-7), 138.7 (1C, C-10), 135.7 (1C, C-4), 129.2 (1C, C-8), 128.6 (2C, C-11, C-11′), 128.5 (2C, C-12, C-12′), 123.3 (1C, C-5), 119.1 (1C, C-6), 114.4 (1C, C-3), 61.3 (1C, C-19), 55.8 (1C, C-18), 37.9 (1C, C-15), 24.5 (1C, C-16), 21.6 (1C, C-14), 13.8 (1C, C-17). HRMS-ESI: *m*/*z* calcd for C_12_H_24_O_3_ (M + H)^+^, 325.1798; found, 325.1795.

##### 4.2.2.2 (*E*)*-*3-(2-(Benzyloxy)-3-methoxy-5-propylphenyl)-1-(*p*-toluyl)­prop-2-en-1-one (**4**)

This product was obtained in 81% yield as a white solid after purification by column chromatography (ethyl acetate/methanol 99.5:0.5 v/v). IR-ATR (cm^–1^): 2956, 2927, 2869 (C–H sp^3^), 1758, 1660 (C=O ketone), 1585, 1452 (Ar. C=C), 1278 (Ar–O–CH_3_). ^1^H NMR (CDCl_3_, 400 MHz): δ 8.01 (d, 1H, ^3^ *J* = 15.92 Hz, H-7), 7.83 (da, 2H, ^3^ *J* = 8.12 Hz, H-11, H-11′), 7.54 (d, 1H, ^3^ *J* = 15.92 Hz, H-8), 7.47 (da, 2H, ^3^ *J* = 8.12 Hz, H-12, H-12′), 7.37–7.32 (m, 3H, H-22, H-22′, H-23), 7.24 (d, 2H, ^3^ *J* = 8.08 Hz, H-21, H-21′), 7.07 (d, 1H; ^4^ *J* = 1.72 Hz, H-4), 6.83 (d, 1H, ^4^ *J* = 1.8 Hz, H-6), 5.03 (s, 2H, H-19), 3.92 (s, 3H, H-18), 2.61 (t, 2H, ^3^ *J* = 7.36 Hz, H-15), 2.44 (s, 3H, H-14), 1.70 (sext, 2H, ^3^ *J* = 7.4 Hz, H-16), 1.00 (t, 3H, ^3^ *J* = 7.28 Hz, H-17). ^13^C NMR (CDCl_3_, 100 MHz): δ 190.7 (1C, C-9), 152.9 (1C, C-3), 145.5 (1C, C-2), 143.2 (1C, C-13), 140.2 (1C, C-7), 138.9 (2C, C-20, C-23), 137.2 (1C, C-10), 135.7 (1C, C-8), 133.0 (1C, C-5), 129.2 (2C, C-11, C-11′), 128.7 (2C, C-12, C-12′), 128.5 (2C, C-21, C-21′), 128.4 (2C, C-22, C-22′), 123.7 (1C, C-6), 119.8 (1C, C-4), 114.6 (1C, C-1), 75.4 (1C, C-19), 55.9 (1C, C-18), 37.9 (1C, C-15), 24.5 (1C, C-16), 21.6 (1C, C-14), 13.8 (1C, C-17). HRMS-ESI: *m*/*z* calcd. for C_27_H_28_O_3_ (M + H)^+^, 401.2171; found, 401.2107.

#### 4.2.3 Synthesis of Compound **6**

In a round-bottom flask was added silica gel 60 (50% hydrated) (3.3 equiv), and subsequently, aldehyde **5** (1 equiv), sodium nitrate (2 equiv), and potassium bisulfate (2.2 equiv) solubilized in dichloromethane (25 mL) were added to the flask. The reaction mixture was vigorously stirred at 25 °C for 5 days. The product was obtained in a pure form after filtration and solvent elimination, without the need for purification.

##### 4.2.3.1 2-Hydroxy-3-methoxy-5-nitrobenzaldehyde (**6**)

This product was obtained in 84% yield as an orange solid without purification. ^1^H NMR (400 MHz, CDCl_3_): δ 10.09 (1H, CHO), 6.58 (d, 1H, ^4^ *J* = 2.4 Hz, H-6), 6.29 (d, 1H, ^4^ *J* = 2.44 Hz, H-4), 2.38 (s, 3H, H-8). ^13^C NMR (100 MHz, CDCl_3_): δ 195.6 (1C, C-7), 157.0 (1C, C-2), 149.2 (1C, C-3), 140.0 (1C, C-5), 120.6 (1C, C-6), 119.0 (1C, C-1), 111.5 (1C, C-4), 57.0 (1C, C-8).

#### 4.2.4 Synthesis of Nitro Chalcone **7**

To a solution containing the 4-methyl-acetophenone (1 equiv) and aldehyde **6** (1 equiv) in ethanol (10 mL) was added 6 mL of an aqueous solution of NaOH (60%) dropwise over 30 min under magnetic stirring. The mixture was kept under stirring for 4 h at 25 °C when the reaction was completed. The reaction mixture was neutralized, under cooling, using aqueous 1 M HCl, and the obtained solid was collected by filtration. The product was obtained in a pure form, without the need for purification.

##### 4.2.4.1 (*E*)-3-(2-Hydroxy-3-methoxy-5-nitrophenyl)­1-(*p*-tolyl)­prop-2-en-1-one (**7**)

This product was obtained in 91% yield as a yellow solid without purification. MP: 128–132 °C. IR-ATR (cm^–1^): 3244, 2972, 2839, 1660, 1585, 1517, 1319, 1249, 1176, 1072, 864, 761. ^1^H NMR (400 MHz, DMSO-*d*_6_): δ 8.40 (d, 1H, ^4^ *J* = 2.6 Hz, H-6), 8.09–7.97 (m, 4H, H-7, H-8, H-11, H-11′), 7.59 (d, 1H, ^4^ *J* = 2.6 Hz, H-4), 7.37 (d, 2H, ^3^ *J* = 8.0 Hz, H-12, H-12′), 3.87 (s, 3H, H-15), 2.40 (s, 3H, H-14).^13^C NMR (100 MHz, DMSO-*d*_6_): δ 195.6 (1C, C-7), 157.0 (1C, C-2), 149.2 (1C, C-5), 140.0 (1C, C-1), 120.6 (1C, C-6), 111.5 (1C, C-3), 57.0 (1C, C-8). HRMS (ESI): calcd for C_17_H_15_NO_5_ [M + Na]^+^, 336.0842; found, 336.0846.

#### 4.2.5 Synthesis of Compound **8**

Chalcone **7** (1 equiv) was solubilized in ethanol (15 mL), hydrated tin chloride (5 equiv) was added to this solution, and the mixture was subjected to stirring at 70 °C for 2 h. The solvent was eliminated, water (10 mL) was added to the flask, and pH was neutralized using an 8% solution of sodium bicarbonate. The resulting solution was extracted with ethyl acetate (3 × 50 mL), and the organic phase was washed with water (2 × 100 mL), dried with anhydrous Na_2_SO_4_, and concentrated. The product was obtained in a pure form, without the need for purification.

##### 4.2.5.1 (*E*)-3-(5-Amino-2-hydroxy-3-methoxyphenyl)-1-(*p*-tolyl)­prop-2-en-1-one (**8**)

This product was obtained in 51% yield as a red solid without purification. MP: 76–80 °C. IR-ATR (cm^–1^): 3361, 3226, 2921, 1650, 1589, 1475, 1444, 1286, 1193, 1083, 991, 815, 779, 607, 590. ^1^H NMR (400 MHz, DMSO-*d*_6_): δ 8.40 (d, 1H, ^4^ *J* = 2.64 Hz, H-6), 8.09–7.97 (m, 4H, H-7, H-8, H-11, H-11′), 7.59 (d, 1H, ^4^ *J* = 2.64 Hz, H-4), 7.37 (d, 2H, ^3^ *J* = 8.0 Hz, H-12, H-12′), 3.87 (s, 3H, H-15), 2.40 (s, 3H, H-14). ^13^C NMR (100 MHz, DMSO-*d*_6_): δ 195.67 (1C, C-7), 157.07 (1C, C-2), 149.25 (1C, C-5), 140.06 (1C, C-1), 120.64 (1C, C-6), 111.57 (1C, C-3), 57.00 (1C, C-8). HRMS (ESI): calcd for C_17_H_15_NO_3_ [M + H]^+^, 284.1281; found, 284.1278.

#### 4.2.6 General Procedure for the Synthesis of Compounds **10** and **11**

To a solution containing the corresponding acetophenone (1 equiv) and formyl-dihydroeugenol **9** (1 equiv) in ethyl alcohol (10 mL) was added 6 mL of an aqueous solution of NaOH (60%) dropwise over 30 min, under magnetic stirring. The mixture was kept under stirring for 4 h at 25 °C until the total consumption of the starting reagents was achieved. The reaction mixture was neutralized, under cooling, using an aqueous 1 M HCl, and the obtained solid was collected by filtration. The product was obtained in a pure form, without the need for purification.

##### 4.2.6.1 (*E*)*-*3-(2-Hydroxy-3-methoxy-5-propylphenyl)-1-(4-hydroxyphenyl)­prop-2-en-1-one (**10**)

This product was obtained in 32% yield as a red solid. IR-ATR (cm^–1^): 3359, 3174, 2958, 2921, 2869, 2848, 1731, 1637, 1587, 1558, 1490, 1436, 1321, 1236, 1166, 1108, 983, 825, 617. MP: 82–92 °C. ^1^H NMR (400 MHz, DMSO-*d*_6_): δ 8.05–8.00 (m, 3H, H-11, H-7), 7.79 (d, 1H, ^3^ *J* = 15.5 Hz, H-8), 7.31 (s, 1H, H-6), 6.90–6.86 (m, 3H, H-12, H-4), 3.82 (s, 3H, H-17), 2.5 (s, 2H, H-14), 1.66–1.59 (m, 2H, H-15), 0.92 (t, 3H, ^3^ *J* = 7.2 Hz, H-16). ^13^C NMR (100 MHz, DMSO-*d*_6_): δ 187.3 (1C, C-9), 162.0 (1C, C-13), 147.8 (1C, C-3), 144.4 (1C, C-2), 138.1 (1C, C-7), 132.9 (1C, C-5), 130.9 (2C, C-11), 129.3 (1C, C-10), 121.3 (1C, C-8), 120.7 (1C, C-1), 118.7 (1C, C-6), 115.3 (2C, C-12), 113.7 (1C, C-4), 55.9 (1C, C-17), 37.0 (1C, C-14), 37.0 (1C, C-14), 24.3 (1C, C-15), 13.7 (1C, C-16). HRMS (ESI): calcd for C_19_H_20_O_4_ [M + Na]^+^, 335.1254; found, 335.1257.

##### 4.2.6.2 (*E*)-3-(2-Hydroxy-3-methoxy-5-propylphenyl)-1-phenylprop-2-en-1-one (**11**)

This product was obtained in 82% yield as a red solid. MP: 58–62 °C. IR-ATR (cm^–1^): 3344, 2945, 2927, 2864, 1649, 1581, 1566, 1488, 1461, 1433, 1274, 1207, 1151, 1093, 985, 837, 784, 690. MP: 58–62 °C. ^1^H NMR (400 MHz, CDCl_3_): δ 8.04–7.99 (m, 3H, H-7, H-11), 7.73 (d, 1H, ^3^ *J* = 15.8 Hz, H-8), 7.58–7.54 (m, 1H, H-13), 7.51–7.47 (m, 2H, H-12), 6.98 (d, 1H, ^4^ *J* = 1.5 Hz, H-6), 6.72 (d, 1H, ^4^ *J* = 1.5 Hz, H-4), 6.13 (sl, 1H, OH), 3.91 (s, 3H, H-17), 2.54 (t, 2H, ^3^ *J* = 7.4 Hz, H-14), 1.64 (sext, 2H, ^3^ *J* = 7.6 Hz, H-15), 0.95 (t, 3H, ^3^ *J* = 7.3 Hz, H-16). ^13^C NMR (100 MHz, DMSO-*d*_6_): δ 191.6 (1C, C-9), 146.9 (1C, C-3), 144.1 (1C, C-2), 140.7 (1C, C-7), 138.8 (1C, C-10), 134.3 (1C, C-5), 132.7 (1C, C-13), 128.8 (2C, C-12), 128.7 (2C, C-11), 123.6 (1C, C-8), 121.4 (1C, C-6), 121.0 (1C, C-1), 112.8 (1C, C-4), 56.4 (1C, C-17), 37.9 (1C, C-14), 24.9 (1C, C-15), 14.0 (1C, C-16). HRMS (ESI): calcd for C_19_H_20_O_3_ [M + Na]^+^, 319.1304; found, 319.1297.

#### 4.2.7 Synthesis of Dimer Chalcone **12**

To a solution containing the 1,4-diacetylbenzene (1 equiv) and formyl-dihydroeugenol **9** (2 equiv) in ethyl alcohol (10 mL) was added 6 mL of an aqueous solution of NaOH (60%) dropwise over 30 min under magnetic stirring. The mixture was kept under stirring for 4 h at 25 °C until the total consumption of the starting reagents was achieved. The reaction mixture was neutralized, under cooling, using an aqueous 1 M HCl, and the obtained solid was collected by filtration. The product was obtained in a pure form, without the need for purification.

##### 4.2.7.1 (2*E*,2′*E*)-1,1′-(1,4-Phenylene)­bis­(3-(2-hydroxy-3-methoxy-5-propylphenyl)­prop-2-en-1-one) (**12**)

This product was obtained in 98% yield as a yellow solid. IR-ATR (cm^–1^): 3338, 2958, 2927, 2869, 1650, 1581, 1488, 1433, 1274, 1245, 1211, 1151, 1091, 991, 829. MP: 122–132 °C. ^1^H NMR (400 MHz, DMSO-*d*_6_): δ 8.23 (s, 4H, H-11), 8.11 (d, 2H, ^3^ *J* = 15.7 Hz, H-7), 7.83 (d, 2H, ^3^ *J* = 15.7 Hz, H-8), 7.32 (d, 2H, ^4^ *J* = 1.5 Hz, H-6), 6.89 (d, 2H, ^4^ *J* = 1.5 Hz, H-4), 3.82 (s, 6H, H-15), 2.53–2.49 (m, 4H, H-12), 1.62 (sext, 4H, ^3^ *J* = 7.6 Hz, H-13), 0.92 (t, 6H, ^3^ *J* = 7.2 Hz, H-14). ^13^C NMR (100 MHz, DMSO-*d*_6_): δ 189.2 (2C, C-9), 147.9 (2C, C-3), 145.4 (2C, C-2), 140.9 (2C, C-10), 140.4 (2C, C-7), 132.7 (2C, C-5), 128.6 (4C, C-11), 120.9 (2C, C-8), 120.6 (2C, C-1), 119.0 (2C, C-6), 114.3 (2C, C-4), 55.9 (2C, C-15), 36.9 (2C, C-12), 24.2 (2C, C-13), 13.7 (2C, C-14). HRMS (ESI): calcd for C_32_H_34_O_6_ [M + Na]^+^, 537.2247; found, 537.2231.

#### 4.2.8 Synthesis of *C*-Acetyl Derivative **14**

The dihydroeugenol **13** (1.1 equiv) was solubilized in anhydrous dichloromethane (6 mL), and to this solution was added anhydrous aluminum chloride (2.5 equiv); this mixture was kept under cooling at 0 °C under magnetic stirring. Then, acetyl bromide (1.0 equiv) was added, and after this addition, the reaction was kept under stirring at 25 °C overnight under a nitrogen atmosphere. After 24 h, water was added to the reaction mixture, which was filtered, and the filtrate obtained was extracted with dichloromethane (3 × 20 mL). The obtained organic phase was washed with 1 M HCl (3 × 25 mL) and water (2 × 100 mL), and then, it was dried with anhydrous sodium sulfate; the solvent was eliminated. The crude product obtained was purified on a silica column (hexane/ethyl acetate 95:5), affording derivative **14** (37% yield).

##### 4.2.8.1 2-Acetyl-6-methoxy-4-propyl-phenol (**14**)

This product was obtained in 37% yield as a yellow solid. ^1^H NMR (400 MHz, CDCl_3_): δ 7.30 (s, 1H, H-6), 6.69 (s, 1H, H-4), 3.93 (s, 3H, H-12), 2.83–2.80 (m, 2H, H-7), 2.52 (s, 3H, H-11), 1.61–1.52 (m, 2H, H-8), 0.96 (t, 3H, ^3^ *J* = 7.3 Hz, H-9). ^13^C NMR (100 MHz, CDCl_3_): δ 200.3 (1C, C-10), 149.02 (1C, C-3), 142.9 (1C, C-2), 137.4 (1C, C-5), 130.2 (1C, C-1), 116.4 (1C, C-6), 113.3 (1C, C-4), 56.1 (1C, C-12), 36.4 (1C, C-7), 29.8 (1C, C-11), 25.3 (1C, C-8), 14.4 (1C, C-9).

#### 4.2.9 General Procedure for the Synthesis of Retroisosteric Chalcones **15**–**18**

To a solution containing the *C*-acetyl-dihydroeugenol **14** (1 equiv) and the corresponding aldehyde (1 equiv) in ethyl alcohol (10 mL) was added 6 mL of an aqueous solution of NaOH (60%) dropwise over 30 min under magnetic stirring. The mixture was kept under stirring for 4 h at 25 °C until the total consumption of the starting reagents was achieved. The reaction mixture was neutralized, under cooling, using an aqueous 1 M HCl, and the obtained solid was collected by filtration. The product was obtained in a pure form, without the need for purification.

##### 4.2.9.1 (*E*)-1-(2-Hydroxy-3-methoxy-5-propylphenyl)-3-phenylprop-2-en-1-one (**15**)

This product was obtained in 67% yield as a yellow oil. IR-ATR (cm^–1^): 3384, 2956, 2929, 2667, 1658, 1598, 1573, 1510, 1448, 1330, 1269, 1189, 1126, 979, 769, 686, 561. ^1^H NMR (400 MHz, CDCl_3_): δ 7.57–7.55 (m, 2H, H-11), 7.51 (d, 1H, ^3^ *J* = 16.0 Hz, H-8), 7.40–7.38 (m, 3H, H-12, H-9), 7.17–7.13 (m, 2H, H-6, H-13), 6.75 (s, 1H, H-4), 3.95 (s, 3H, H-17), 2.73 (t, 2H, ^3^ *J* = 7.6 Hz, H-14), 1.60 (sext, 2H, ^3^ *J* = 7.6 Hz, H-15), 0.93 (t, 3H, ^3^ *J* = 7.3 Hz, H-16). ^13^C NMR (100 MHz, CDCl_3_): δ 195.1 (1C, C-7), 148.4 (1C, C-3), 145.2 (1C, C-9), 143.0 (1C, C-2), 135.9 (1C, C-10), 135.0 (1C, C-5), 131.7 (1C, C-1), 130.6 (1C, C-13), 129.1 (2C, C-12), 128.6 (2C, C-11), 126.9 (1C, C-6), 115.1 (1C, C-4), 112.8 (1C, C-8), 56.1 (1C, C-17), 35.6 (1C, C-14), 25.5 (1C, C-15), 14.3 (1C, C-16). HRMS (ESI): calcd for C_19_H_20_O_3_ [M + H]^+^, 297.1485; found, 297.1484.

##### 4.2.9.2 (*E*)-1-(2-Hydroxy-3-methoxy-5-propylphenyl)-3-(*p*-tolyl)­prop-2-en-1-one (**16**)

This product was obtained in 80% yield as a yellow oil. IR-ATR (cm^–1^): 3328, 2956, 2925, 2867, 2850, 1658, 1593, 1510, 1444, 1334, 1269, 1126, 981, 810. ^1^H NMR (400 MHz, CDCl_3_): δ 7.50 (m, 3H, H-8, H-11), 7.19 (d, 2H, ^3^ *J* = 7.8 Hz, H-12), 7.10 (d, 2H, H-9, H-6), 6.75 (s, 1H, H-4), 3.94 (s, 3H, H-18), 2.74–2.70 (m, 2H, H-15), 2.37 (s, 3H, H-14), 1.65–1.55 (m, 2H, H-16), 0.92 (t, 3H, ^3^ *J* = 7.3 Hz, H-17). ^13^C NMR (100 MHz, CDCl_3_): δ 195.0 (1C, C-7), 148.1 (1C, C-3), 145.2 (1C, C-2), 142.8 (1C, C-9), 140.9 (1C, C-13), 135.5 (1C, C-5), 132.0 (1C, C-1), 129.6 (2C, C-12), 128.4 (2C, C-11), 125.7 (1C, C-6), 114.9 (1C, C-4), 112.6 (1C, C-8), 55.9 (1C, C-18), 35.4 (1C, C-15), 25.3 (1C, C-16), 21.6 (1C, C-14), 14.1 (1C, C-17). HRMS (ESI): calcd for C_20_H_22_O_3_ [M + Na]^+^, 333.1461; found, 333.1453.

##### 4.2.9.3 (*E*)-1-(2-Hydroxy-3-methoxy-5-propylphenyl)-3-(4-hydroxyphenyl)­prop-2-en-1-one (**17**)

This product was obtained in 43% yield as a yellow solid. MP: 52–58 °C. IR-ATR (cm^–1^): 3620, 3278, 3004, 2950, 2927, 2869, 1622, 1593, 1514, 1440, 1328, 1257, 1151, 979, 844, 817. ^1^H NMR (400 MHz, CDCl_3_): δ 7.45–7.41 (m, 3H, H-8, H-11), 7.09 (s, 1H, H-6), 6.98 (d, 1H, ^3^ *J* = 15.8 Hz, H-9), 6.86 (d, 2H, ^3^ *J* = 8.7 Hz, H-12), 6.74 (s, 1H, H-4), 3.93 (s, 3H, H-17), 2.70 (m, 2H, H-14), 1.59 (sext, 2H, ^3^ *J* = 7.6 Hz, H-15), 0.91 (t, 3H, ^3^ *J* = 7.3 Hz, H-16). ^13^C NMR (100 MHz, CDCl_3_): δ 196.3 (1C, C-7), 158.7 (1C, C-13), 148.3 (1C, C-3), 146.1 (1C, C-9), 143.0 (1C, C-2), 135.6 (1C, C-5), 131.8 (1C, C-10), 130.6 (2C, C-11), 127.3 (1C, C-1), 124.5 (1C, C-6), 116.3 (2C, C-12), 115.1 (1C, C-4), 112.8 (1C, C-8), 56.1 (1C, C-17), 35.5 (1C, C-14), 25.5 (1C, C-15), 14.3 (1C, C-16). HRMS (ESI): calcd for C_19_H_20_O_4_ [M + Na]^+^, 335.1254; found, 335.1255.

##### 4.2.9.4 (*E*)-3-(4-Fluorophenyl)-1-(2-hydroxy-3-methoxy-5-propylphenyl)­prop-2-en-1-one (**18**)

This product was obtained in 31% yield as a yellow oil. IR-ATR (cm^–1^): 3392, 2958, 2933, 2869, 2850, 1660, 1596, 1506, 1413, 1357, 1269, 1126, 981, 827. ^1^H NMR (400 MHz, CDCl_3_): δ 7.57 (m, 2H, H-11), 7.48 (d, 1H, ^3^ *J* = 16.0 Hz, H-8), 7.13 (s, 1H, H-6), 7.11–7.06 (m, 3H, H-9, H-12), 6.75 (s, 1H, H-4), 3.95 (s, 3H, H-17), 2.74–2.71 (m, 2H, H-14), 1.65–1.55 (m, 2H, H-15), 0.95 (t, 3H, ^3^ *J* = 7.3 Hz, H-16). ^13^C NMR (100 MHz, CDCl_3_): δ 194.4 (1C, C-7), 163.9 (1C, d, ^1^ *J* = 250.2 Hz, H-13), 148.2 (1C, C-3), 143.5 (1C, C-9), 142.8 (1C, C-2), 135.7 (1C, C-5), 131.3 (1C, C-1), 131.0 (1C, d, ^4^ *J* = 3.2 Hz, C-10), 130.2 (2C, d, ^3^ *J* = 8.4 Hz, C-11), 126.3 (1C, C-6), 116.0 (2C, d, ^2^ *J* = 21.8 Hz, C-12), 114.9 (1C, C-8), 112.6 (1C, C-4), 55.9 (1C, C-17), 35.4 (1C, C-14), 25.3 (1C, C-15), 14.0 (1C, C-16). HRMS (ESI): calcd for C_19_H_19_FO_3_ [M + Na]^+^, 337.1210; found, 337.1206.

### 4.3 Cytotoxicity Assays

In vitro biological assays were performed by using five human cell lines for cytotoxicity evaluation. HepG2 cells (ATCC HB-8065), derived from a human hepatocellular carcinoma of the liver (15-year-old Caucasian male), were cultured in Dulbecco’s modified Eagle medium (DMEM) supplemented with 5% fetal bovine serum (FBS), penicillin/streptomycin (100 IU/mL), and amphotericin B (5 μg/mL), in a humidified incubator with 5% CO_2_ at 37 °C. These cells were plated at a concentration of 2.0 × 10^4^ cells/well in 96-well microplates. MRC-5 cells (ATCC CCL-171), derived from human lung fibroblasts of a 14-week-old male fetus, were cultured under the same conditions and plated at 1.0 × 10^4^ cells/well. T24 cells (ATCC HTB-4), established from a transitional cell carcinoma of the bladder (81-year-old Caucasian female), were also cultured under identical conditions and plated at a density of 1.0 × 10^4^ cells/well. TOV-21G cells (ATCC CRL-11730), derived from a primary malignant ovarian adenocarcinoma (62-year-old Caucasian female), were maintained in DMEM with the same supplementation and environmental conditions and plated at 5.0 × 10^4^ cells/well. HeLa cells (ATCC CCL-2), originally obtained from the cervical cancer tissue of Henrietta Lacks, an African-American woman, were likewise maintained in DMEM supplemented with 5% FBS, penicillin/streptomycin (100 IU/mL), and amphotericin B (5 μg/mL), in a 5% CO_2_ atmosphere at 37 °C, and plated at a concentration of 5.0 × 10^5^ cells/well.

HeLa, HepG2, MRC-5, T24, and TOV-21G cells were suspended in a culture medium supplemented with 5% fetal bovine serum (FBS) and seeded into 96-well microplates. The plates were incubated under a humidified atmosphere containing 5% CO_2_ at 37 °C for 24 h, allowing for adherence and monolayer formation.^59,60^ Eight serial dilutions of the test samples, previously solubilized in dimethyl sulfoxide (DMSO), were prepared in DMEM supplemented with 1% FBS, covering a concentration range from 100 to 0.78125 μg/mL, and the results were converted to μg/mL. The volume of DMSO in each well was limited to a final concentration not exceeding 2%. Following monolayer formation, the culture medium was aspirated and replaced with 100 μL of the diluted test samples and 100 μL of DMEM containing 1% FBS. The plates were then incubated under the same atmospheric and temperature conditions.

Two hours prior to end point reading, 20 μL of MTT [3-(4,5-dimethylthiazol-2-yl)-2,5-diphenyltetrazolium bromide] solution (1.0 mg/mL in sterile phosphate-buffered saline, Sigma-Aldrich) was added to each well. The incubation period following sample addition varied according to the cell line: 24 h for MRC-5, 48 h for T24, and 72 h for HeLa, HepG2, and TOV-21G. After the appropriate incubation, absorbance was measured by using a VICTOR X3 microplate reader (PerkinElmer), and data were processed with WorkOut 2.5 software at a wavelength of 490 nm. Formazan formation, resulting from the MTT reduction by metabolically active cells, was used as an indirect measure of cell viability. The percentage of cytotoxicity was calculated using the equation % cytotoxicity = [(*A* – *B*)/*A*] × 100, where *A* is the optical density (OD_4__9__0_) of untreated control cells and *B* is the OD_4__9__0_ of treated cells.

Cytotoxicity was expressed as the 50% cytotoxic concentration (CC_5__0_), representing the concentration of sample required to reduce cell viability by 50% relative to that of the untreated control. The experimental design included the following controls: (i) the cell control, consisting of cell monolayers incubated with medium supplemented with 1% FBS only, to assess baseline cell behavior in the absence of any test compounds; (ii) the DMSO control, comprising wells treated with DMSO in the same concentration used to solubilize the samples, to ensure that the solvent itself did not exert cytotoxic effects.

Statistical analysis was performed using GraphPad Prism software, version 7.00. Differences between group means and medians in cytotoxicity and antiviral activity assays were evaluated using Student’s *t* test and one-way analysis of variance (ANOVA). A significance level of 5% (*p* < 0.05) was adopted for all comparisons.

### 4.4 Cell Culture and Solution

The HepG2 (*TP53* wild type) and T24 (*TP53* allele encoding an in-frame deletion of tyrosine 126) cells were cultured for experimentation. These cell lines were selected due to their differing *TP53* gene status, an important tumor suppressor gene frequently mutated in cancer, which plays a key role in regulating cell cycle arrest, DNA repair, and apoptosis. Additionally, they were chosen for representing both metabolizing and nonmetabolizing profiles, allowing for a broader evaluation of the compound’s effects under distinct metabolic and genetic conditions.

The cells were cultured in DMEM (Sigma-Aldrich, St. Louis, USA) and supplemented with 10% fetal bovine serum (Cultilab Ltda., Campinas, Brazil) and antibiotics (amphotericin). They were incubated at 37 °C in a humidified atmosphere with 5% CO_2_. Control cell groups, which were not exposed to the compound, were included in the experiments and were conducted in triplicate. The chalcone was dissolved in dimethyl sulfoxide (DMSO) for the assays. The resulting stock solution had a concentration of 1 mg/mL, which was then diluted to the required concentrations for the experiments.

### 4.5 Clonogenic Survival

The cells were trypsinized and plated into 24-well plates at a density of 1.0 × 10^5^ cells/mL. Afterward, they were treated with the chalcone at concentrations of 2.15, 4.62, 9.25, and 18.50 μM for 48 h. Following treatment, the cells were washed with Hanks’ solution and trypsinized again, and approximately 1000 cells were reseeded into 12-well plates. These cells were incubated for 10 days in a CO_2_ incubator to allow colony formation. After the incubation period, the culture medium was removed, and the cells were washed with Hanks’ solution. Fixation was performed using 4% formaldehyde for 20 min, followed by rehydration in 100% methanol for 20 min. The cells were then stained with a 0.5% crystal violet solution prepared in 25% methanol. Excess stain was removed by washing with a 33% acetic acid solution. Absorbance was measured at 570 nm using a spectrophotometer, and the results were expressed as the percentage of colonies formed relative to the untreated control group.^61^

### 4.6 Cell Migration and Morphology

Cells were seeded in 24-well plates at a density of 1.0 × 10^5^ cells per well and incubated for 24 h to allow attachment. After this incubation, a sterile 200 μL pipet tip was used to scrape the cell monolayer, creating a wound. The initial wound area was photographed using an inverted microscope equipped with a camera. Following this, the cells were washed with Hanks’ solution and treated with the glycosylated chalcone at concentrations of 2.15, 4.62, 9.25, and 18.50 μM for 48 h. At the end of the treatment period, the cells were photographed again, and migration into the wound area was assessed. The percentage of cell migration was quantified using ImageJ and GraphPad Prism software.^62^

For the morphology assay, 2.0 × 10^5^ cells were cultured in 12-well plates for 24 h to allow adherence. After this period, the cells were treated with the chalcone at concentrations of 2.15, 4.62, 9.25, and 18.50 μM for 48 h. After treatment, the cells were examined and photographed under an inverted microscope at 200× magnification.

### 4.7 Cell Cycle

Cells were seeded in 12-well plates at a density of 1.0 × 10^5^ cells per well and incubated for 24 h to allow adhesion. Following this initial incubation, the cells were treated with the chalcone at concentrations of 0.92, 2.15, 4.62, and 9.25 μM for an additional 48 h. After the treatment, the cells were washed, trypsinized, and collected along with the culture supernatant into Falcon tubes. The samples were then centrifuged at 1000 rpm for 10 min. After centrifugation, the supernatant was discarded, and the cell pellets were resuspended in 200 μL of propidium iodide staining solution before being transferred to cytometry tubes. These tubes were incubated on ice, in the dark, for 30 min. Flow cytometric analysis was performed on 20,000 events using a BD FACScalibur flow cytometer (BD Biosciences, Franklin Lakes, NJ, USA), and the data were analyzed using FlowJo software.^27^

### 4.8 Apoptosis Assay

Cells in the exponential growth phase were seeded in 12-well plates at a density of 1 × 10^5^ cells per well in 3 mL of complete culture medium. Along with the experimental groups, four control groups were included: one without labeling, one labeled only with propidium iodide (PI), one labeled only with Annexin V, and one labeled with both PI and Annexin V. After 24 h, the cells were treated with the compound at concentrations of 2.15, 4.62, 9.25, and 18.50 μM for 48 h. Following the treatment period, the supernatant from each well was transferred to cytometry tubes and centrifuged at 1100 rpm for 10 min. The remaining adhered cells were washed with 1 mL of Hanks’ solution, and 500 μL of trypsin was added to detach the cells. The trypsin action was neutralized by adding 500 μL of complete medium, and the contents (detached cells plus precentrifuged supernatant) were collected into cytometry tubes, which were then centrifuged again at 1100 rpm for 10 min. After centrifugation, the supernatant was discarded. For labeling, Annexin V binding buffer^1^ was prepared by diluting 1 mL of component C^5^ in 5 mL of distilled water. Next, a 100 μg/mL propidium iodide working solution was prepared by diluting 5 μL of the 1 mg/mL stock (component B) in 45 μL of 1× buffer. In each sample, cells were resuspended in 100 μL of 1× buffer, and then, 5 μL of FITC-conjugated Annexin V (component A) and 1 μL of PI solution were added. The tubes were briefly vortexed and incubated for 15 min at room temperature, protected from light. After incubation, another 100 μL of 1× buffer was added, and the samples were quickly homogenized and kept on ice until flow cytometry data acquisition.

### 4.9 Statistical Analyses

All experiments were conducted in triplicate, and the resulting data were analyzed using GraphPad Prism 7.0. The normal distribution of the presented data was verified by using the Shapiro–Wilk test. The results of the assays for clonogenic survival, migration, cell cycle, and apoptosis were analyzed with one-way ANOVA followed by a Dunnett post-test to compare with the control group. Significance was considered for a *p*-value of less than 0.05.

### 4.10 Computational Details

Physicochemical descriptors and pharmacokinetic properties of chalcone **3** were predicted with the SwissADME Web server (http://www.swissadme.ch/) using its SMILES as input. [REF SwissADME] Target prediction based on 2D and 3D ligand similarity was performed with SwissTargetPrediction (https://www.swisstargetprediction.ch/), also using the SMILES code [REF SwissTargetPrediction].

Molecular modeling studies were carried out using MMP-9, a metalloproteinase of the gelatinase family responsible for extracellular matrix degradation.^63,64^ This enzyme was selected as a model due to its well-established role in tumor progression and the availability of structural data.^65–67^ The crystallographic structure used in the studies (PDB code 4xct) was complexed with a hydroxamic acid inhibitor, with both oxygens of the hydroxamate group coordinated with catalytic Zn^2+^ (distances of 2.16 and 2.31 Å).^31^

Chalcone **3** and the crystallographic inhibitor tridimensional structures were generated in Open Babel.^68^ A conformer search was conducted, and the lowest-energy conformer was further minimized with 50,000 steps of the steepest descent algorithm using the MMFF94 force field.

Prior to MD, docking was performed in GOLD to generate the complex.^69^ The protein was prepared by removing water and all other molecules, with the exception of Zn^2+^ and Ca^2+^ ions. For each ligand, 100 independent docking runs, with a minimum of 150,000 operations in a search area delimited to residues within 6 Å of the crystallographic ligand, were conducted using the ChemPLP scoring function. Redocking of the crystallographic inhibitor with an RMSD of 1.34 Å validated the protocol.^70^

MD simulations were performed on Gromacs with the best pose obtained on docking to analyze complex stability.^71^ Ligand topology was generated in Acpype using the GAFF2 force field, while Amber99SB-ILDN was used from the protein, with additional parameters to account for His and Asp coordination with zinc atoms.^67,72,73^ The system was solvated using the TIP3P water model and neutralized with Na^+^ and Cl^–^ ions at a final concentration of 0.15 M. Energy minimization was followed by equilibration for 1000 ps under NVT and NPT ensembles at 310 K and 1 atm by using the V-rescale and C-rescale algorithms, respectively. The production phase initially consisted of a 25 ns of simulation with a 2 fs time step using the leapfrog algorithm under periodic boundary conditions. Long-range electrostatics and van der Waals interactions were treated using the particle mesh Ewald (PME) method, with a cutoff of 1.0 nm and a potential-shift modifier.^74^ The LINCS algorithm was used to constrain hydrogen bonds. Trajectories were analyzed by using built-in Gromacs tools to calculate RMSD and Zn–O interatomic distances.

## Supplementary Material



## Notes

^1^H, ^13^C NMR, infrared, and HRMS spectra and molecular modeling data of synthesized compounds (PDF)
