# Supplementary material for: Design and Synthesis of Novel Chalcone Derivatives Targeting Bladder and Liver Cancer: Cytotoxicity Evaluation and Preliminary Mechanistic Studies
Source: ACS Omega. 2025 Dec 2;10(49):60612–26. doi: 10.1021/acsomega.5c08480 (PMC12713470; doi:10.1021/acsomega.5c08480)

Design and synthesis of novel chalcone derivatives targeting bladder and liver cancer: cytotoxicity evaluation and preliminary mechanistic studies

Aline Mol Hermenegildo[a], Izadora Amaral Nakao[a], Luana Beatriz Araújo Vaz[a], Gabrielly Guimarães Coutinho[a], João Lucas Moraes Toledo[a], Tamires Cunha Almeida[b], Tatiane Roquete Amparo[a], Kamila de Fatima Anunciação[a], Glenda Nicioli da Silva[a], Geraldo Célio Brandão[a], Saulo Fehelberg Pinto Braga[a], Thiago Belarmino de Souza[a,*]

[a] *School of Pharmacy - Federal University of Ouro Preto, 35402-163, Ouro Preto, MG, Brazil.*

[b] *Center for Development and Innovation - Butantan Institute,* 05509-002, *Sao Paulo, SP, Brazil.*

*Corresponding author:
E-mail: thiago.souza@ufop.edu.br
phone: +55.35.99829.0199
Mailing address:
Universidade Federal de Ouro Preto
(Campus Morro do Cruzeiro – Escola de Farmácia)
City/State: Ouro Preto/MG; Brazil
Zip code: 35402-163

Contents:

Physicochemical and pharmacokinetic data of chalcone **3** (Table S1).

Representative ^{1}H and ^{13}C NMR, infrared, high resolution mass spectra of synthesized compounds (Figures S1-S48)

Table S1 – Physicochemical descriptors and pharmacokinetic properties of chalcone **3** predicted with SwissADME.

**Physicochemical properties**	
Formula	$C_{21}H_{24}O_3$
Molecular weight	324.41 g/mol
Number of heavy atoms	24
Aromatic heavy atoms	12
Fraction of Csp3	0.29
Rotatable bonds	7
H-bond acceptors	3
H-bond donors	0
Molar refractivity	98.78
TPSA	35.53
**Lipophilicity ($logP_{o/w}$)**	
iLOGP	3.86
XLOGP3	5.34
WLOGP	4.75
MLOGP	3.58
Silicos-IT LogP	5.86
Consensus LogP	4.68
**Water solubility**	
ESOL LogS	-5.12
ESOL Solubility (mg/ml)	2.44e-03
ESOL Class	*Moderately soluble*
Ali LogS	-5.84
Ali Solubility (mg/ml)	4.70e-04
Ali Class	*Moderately soluble*
Silicos-IT LogS	-6.77
Silicos-IT Solubility (mg/ml)	5.52e-05
Silicos-IT class	*Poorly soluble*
**Pharmacokinetics**	
GI absorption	*High*
BBB permeant	*Yes*
Pgp substrate	*No*
CYP1A2 inhibitor	*Yes*
CYP2C19 inhibitor	*Yes*
CYP2C9 inhibitor	*No*
CYP2D6 inhibitor	*Yes*
CYP3A4 inhibitor	*Yes*
log Kp (skin penetration)	-4.49 cm/s
**Druglikeness**	
Lipinski violations	0
Ghose violations	0
Veber violations	0
Egan violations	0

Muegge violations	1 (XLOGP3 > 5)
Bioavailability Score	0.55

Figure S1 – High resolution mass spectrum of compound 2

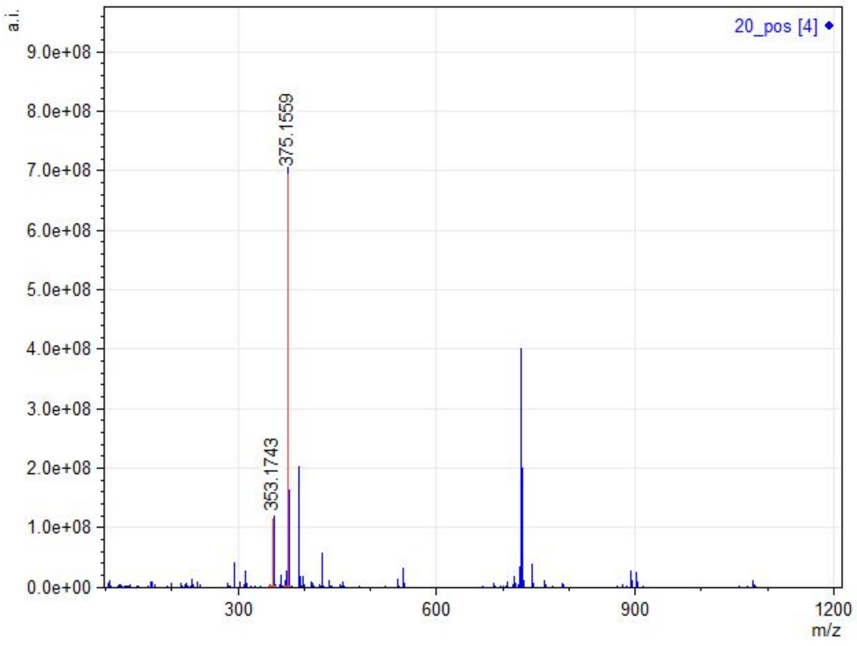

Figure S2 – ^{13}C NMR spectrum of compound 2

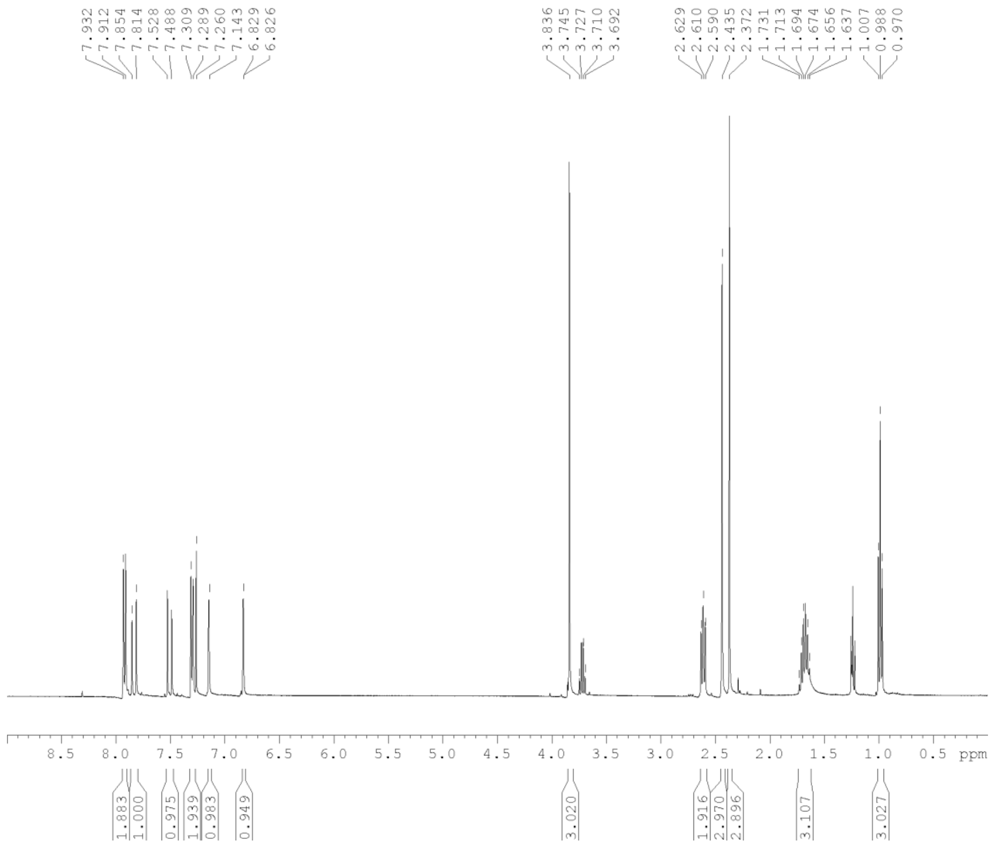

Figure S3 – ^{13}C NMR spectrum of compound 2

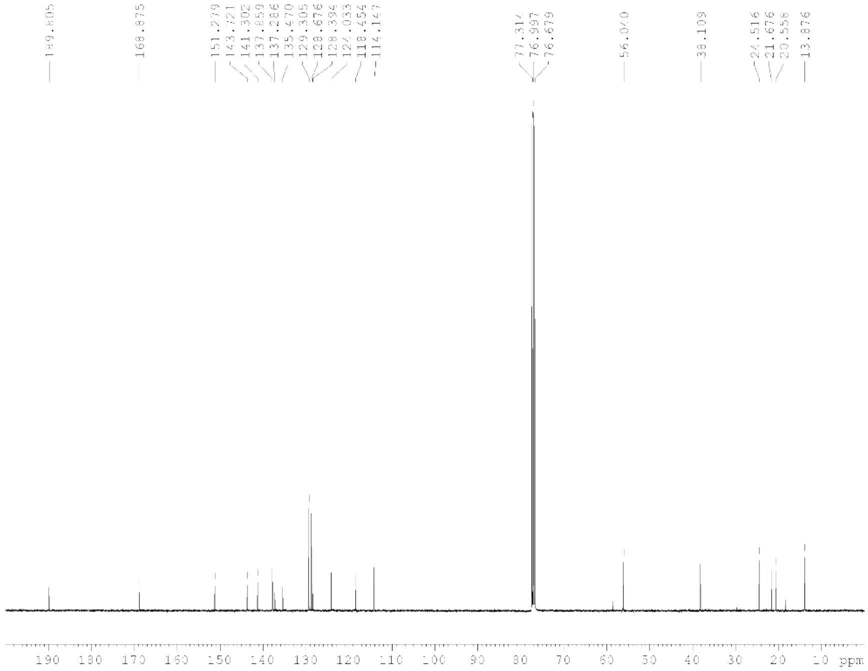

Figure S4 – Infrared spectrum of compound 2

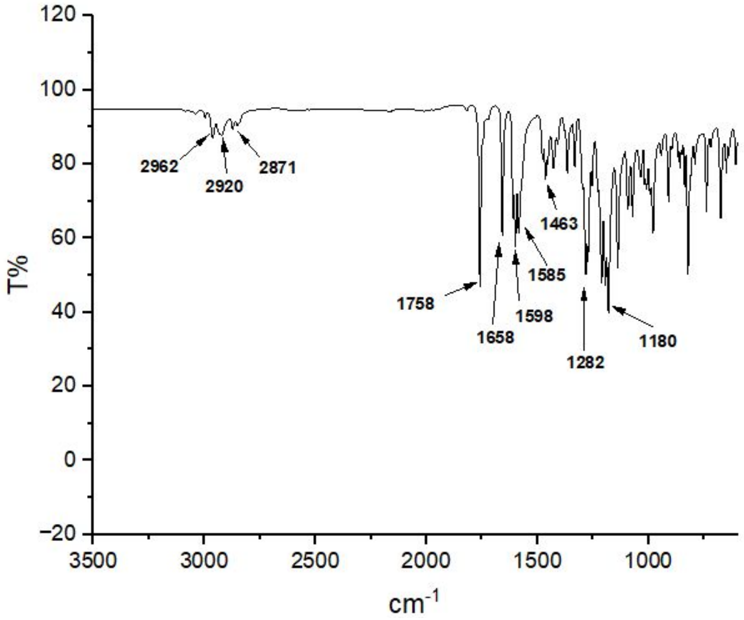

Figure S5 – High resolution mass spectrum of compound 3

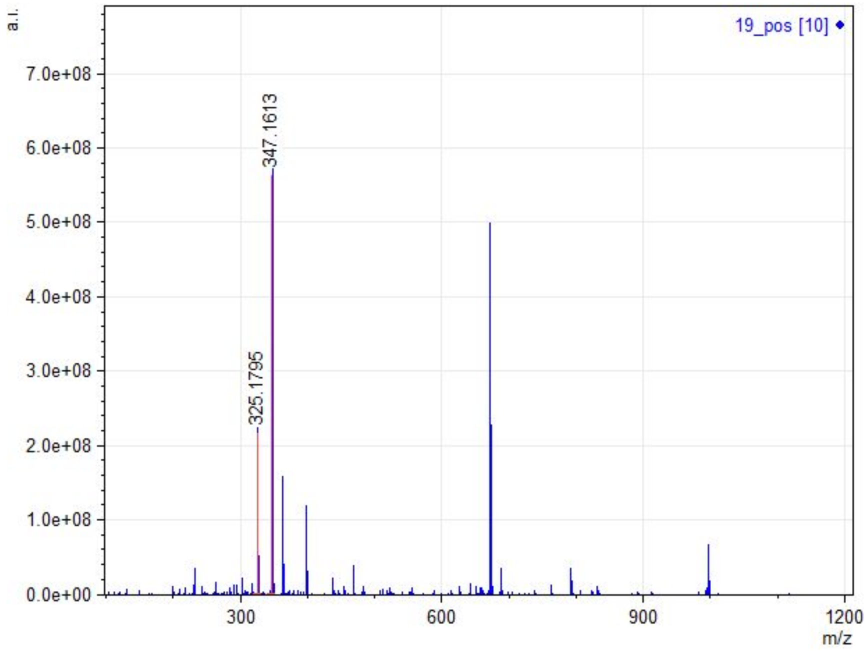

Figure S6 – ^{1}H NMR spectrum of compound 3

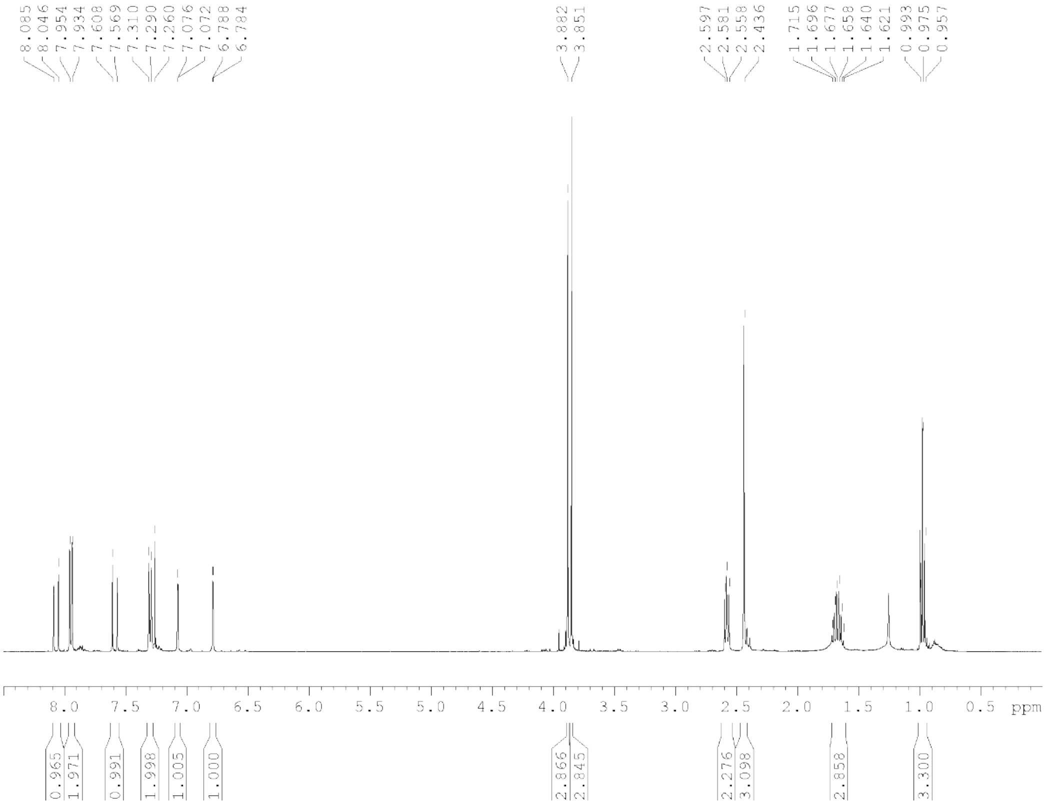

Figure S7 – ^{13}C NMR spectrum of compound 3

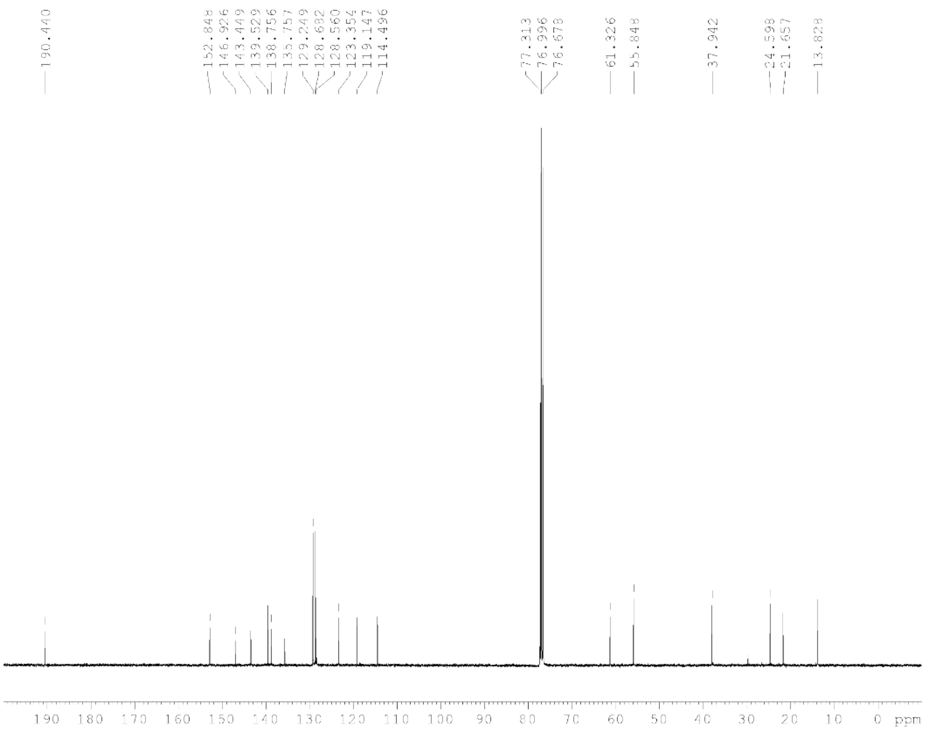

Figure S8 – Infrared spectrum of compound 3

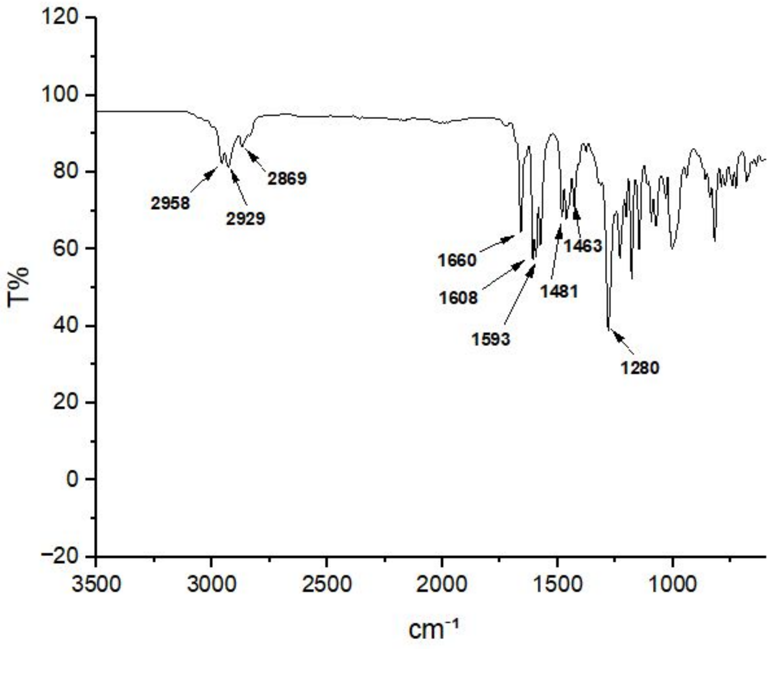

Figure S9 – High resolution mass spectrum of compound 4

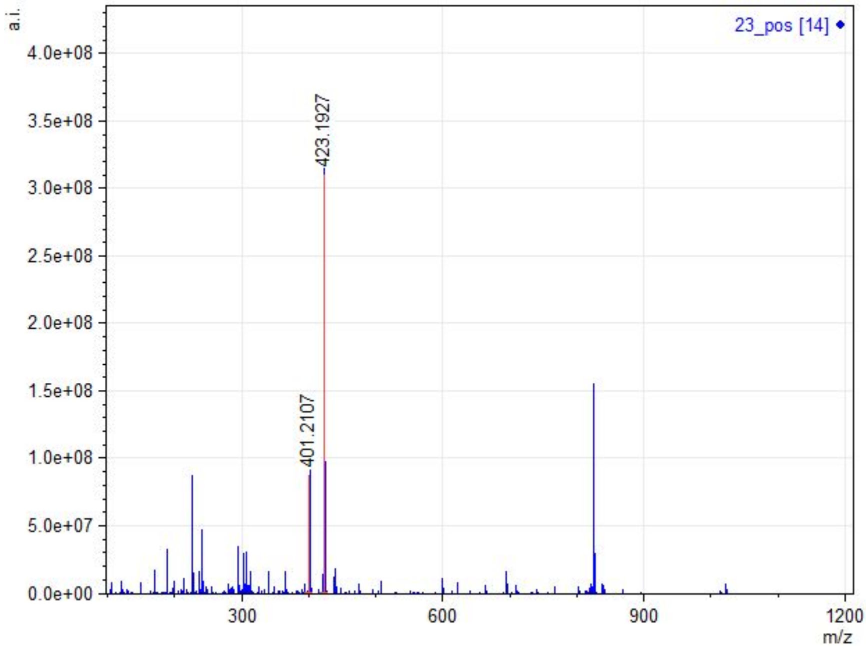

Figure S10 – ^{1}H NMR spectrum of compound 4

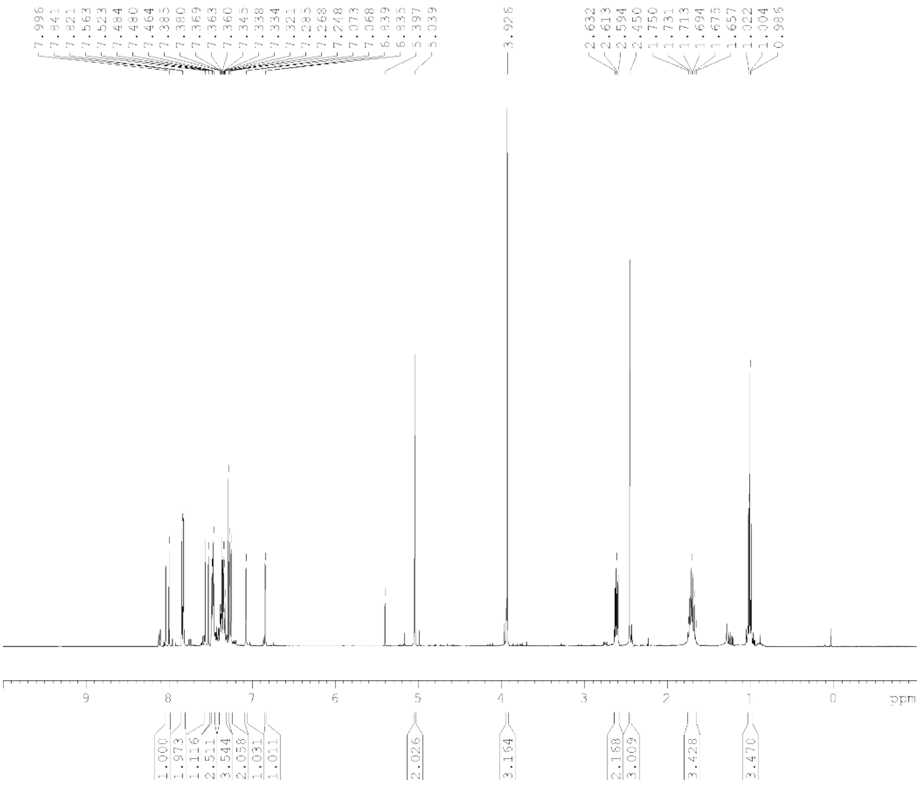

Figure S11 – ^{13}C NMR spectrum of compound 4

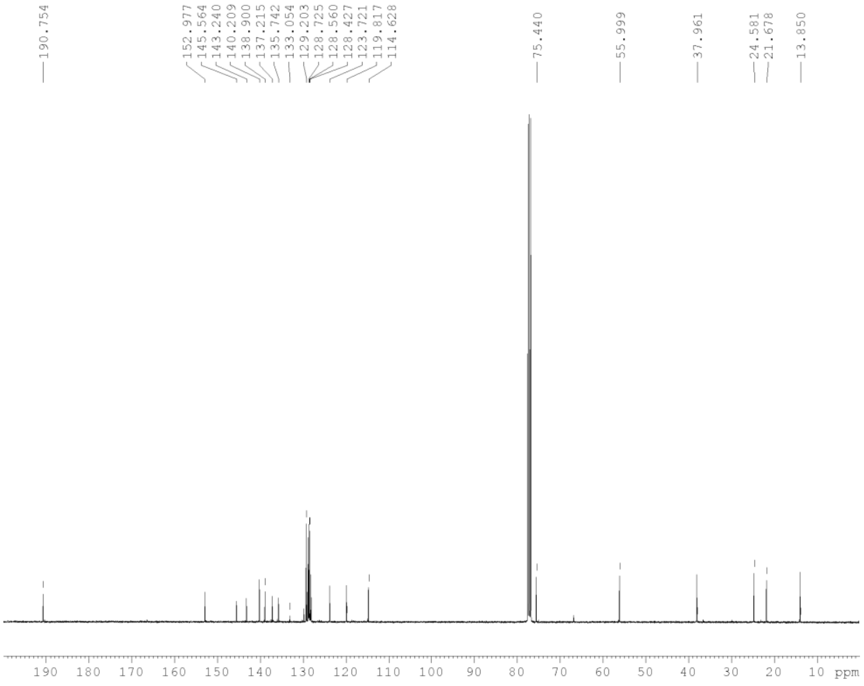

Figure S12 – Infrared spectrum of compound 4

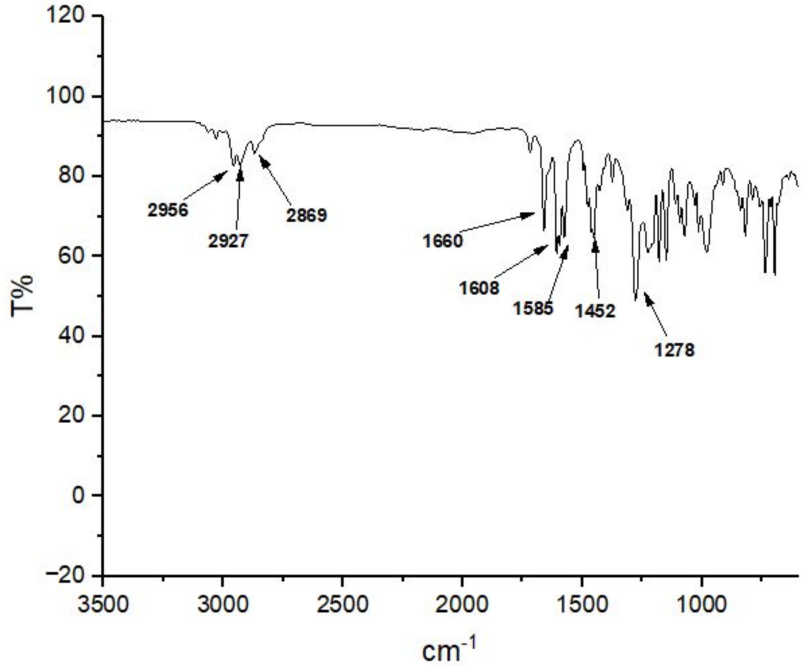

Figure S13 – High resolution mass spectrum of compound 7

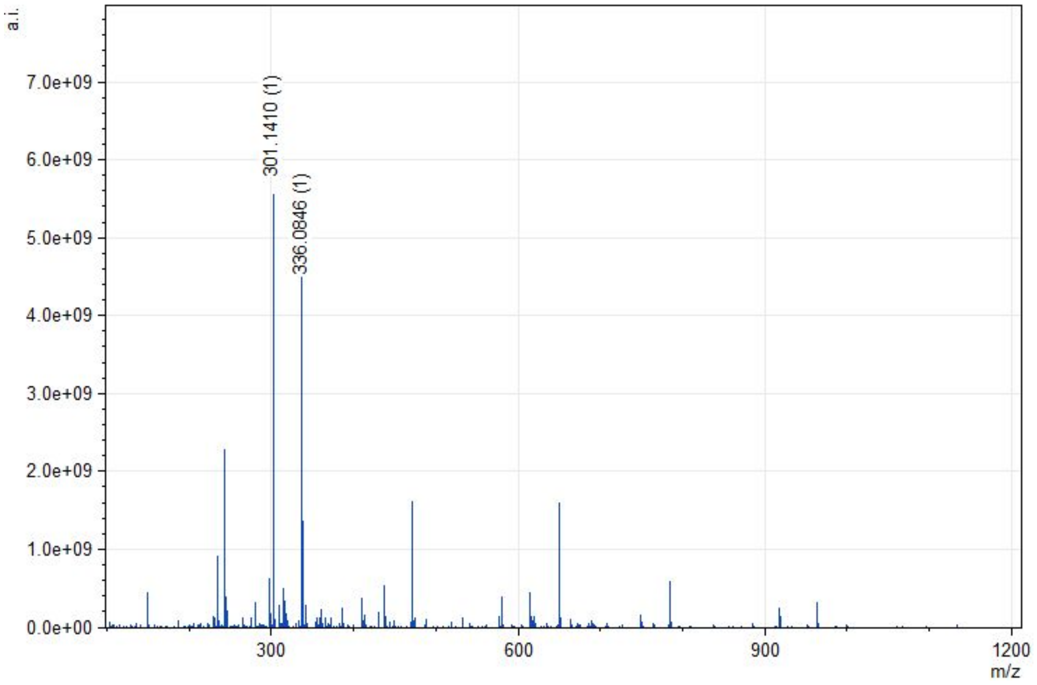

Figure S14 – ^{1}H NMR spectrum of compound 7

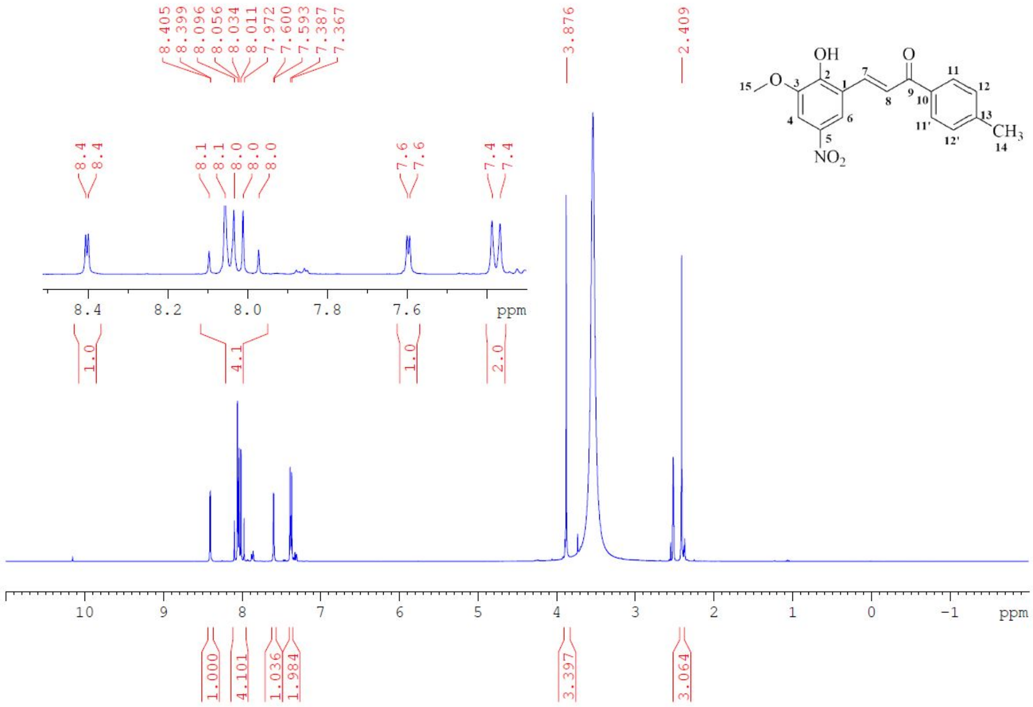

Figure S15 – ^{13}C NMR spectrum of compound 7

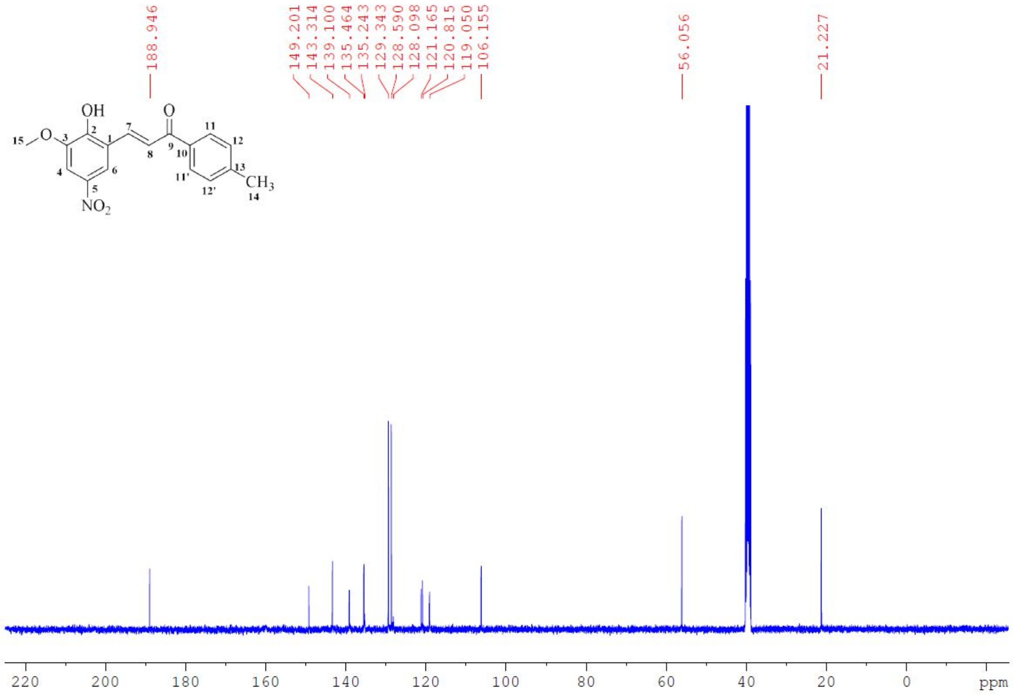

Figure S16 – Infrared spectrum of compound 7

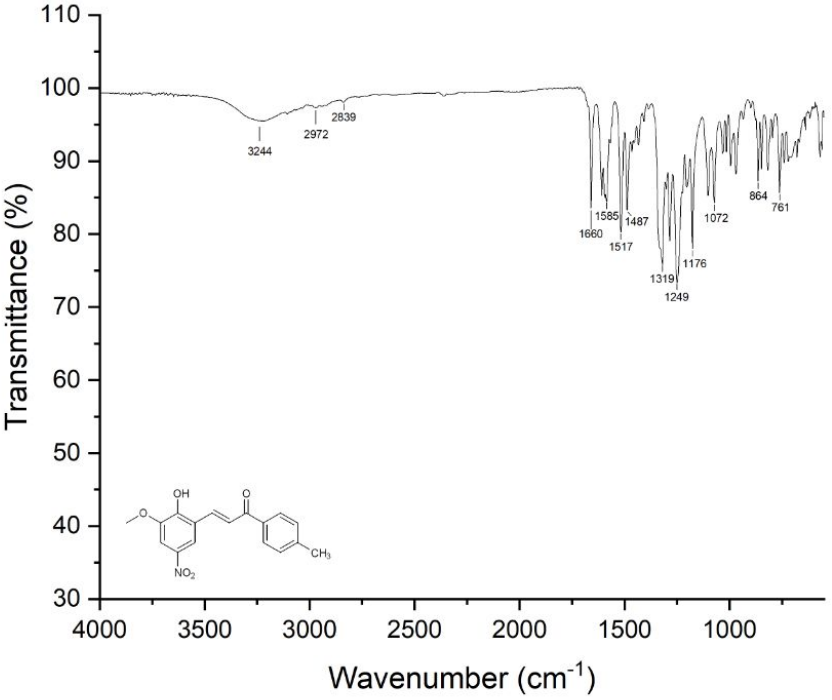

Figure S17 – High resolution mass spectrum of compound 8

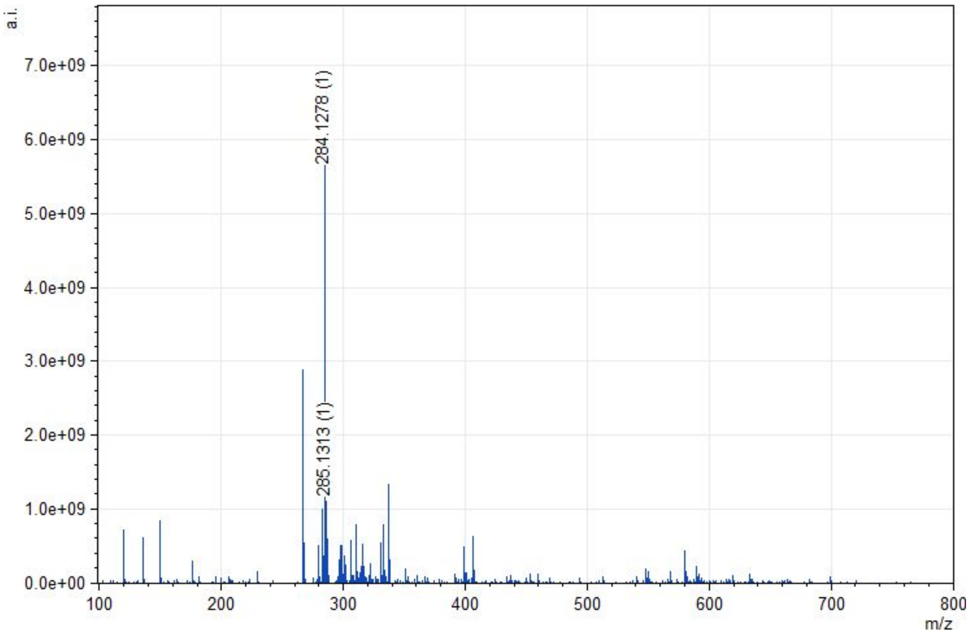

Figure S18 – ^{1}H NMR spectrum of compound 8

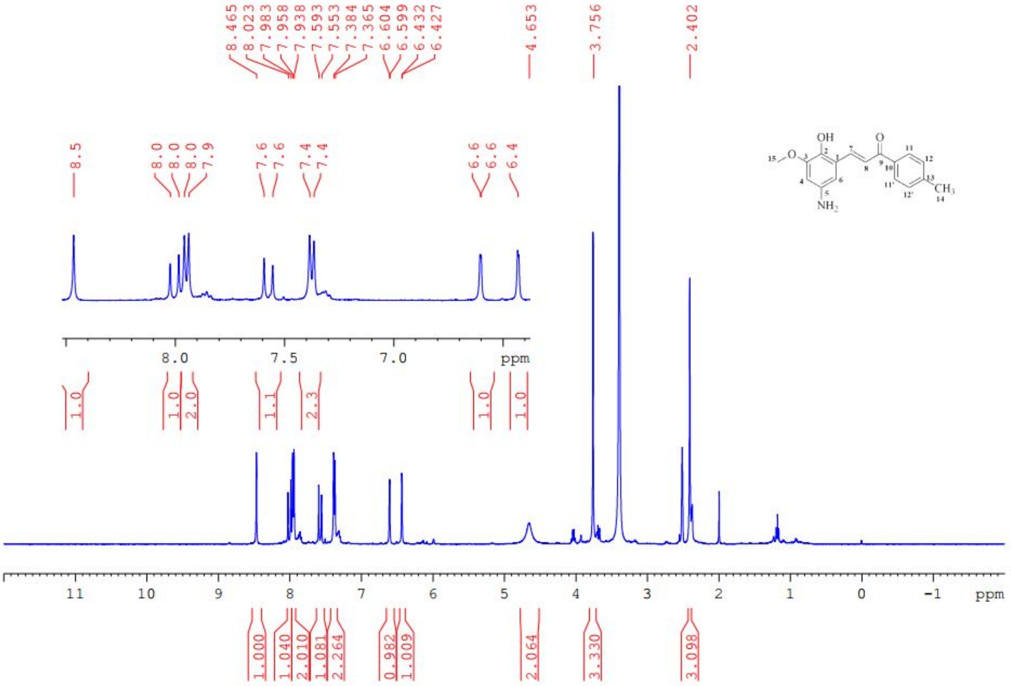

Figure S19 – ^{13}C NMR spectrum of compound 8

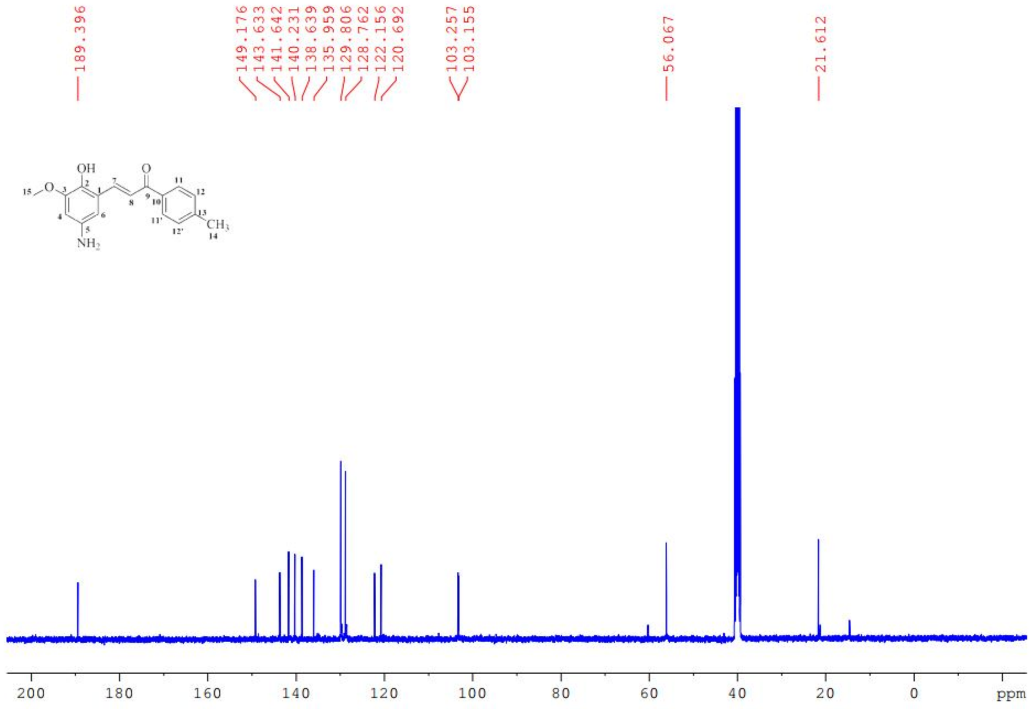

Figure S20 – Infrared spectrum of compound 8

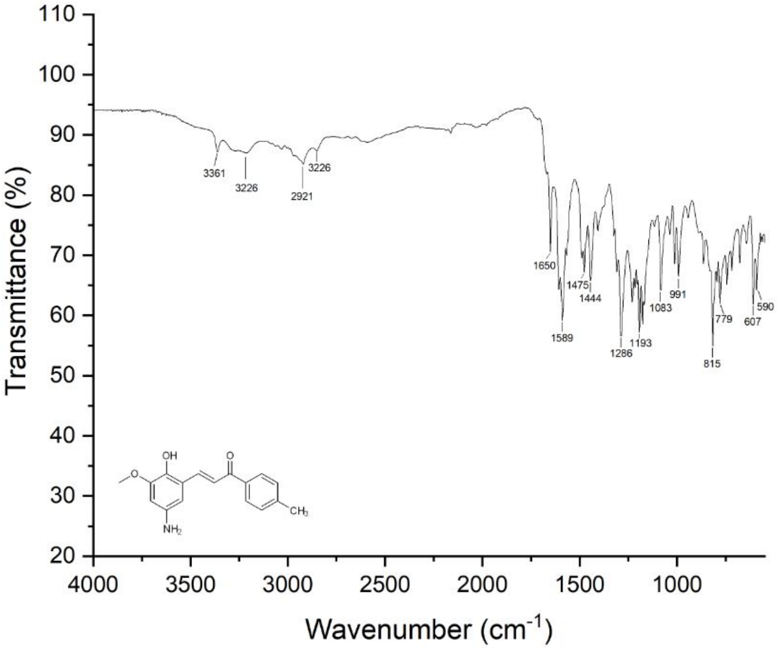

Figure S21 – High resolution mass spectrum of compound 10

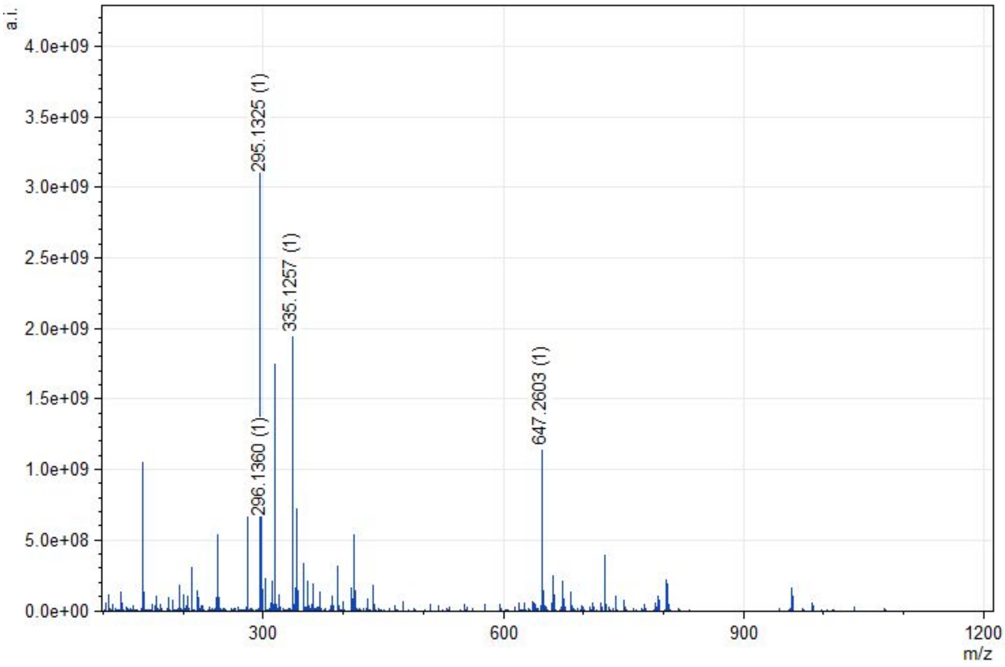

Figure S22 – ^{1}H NMR spectrum of compound 10

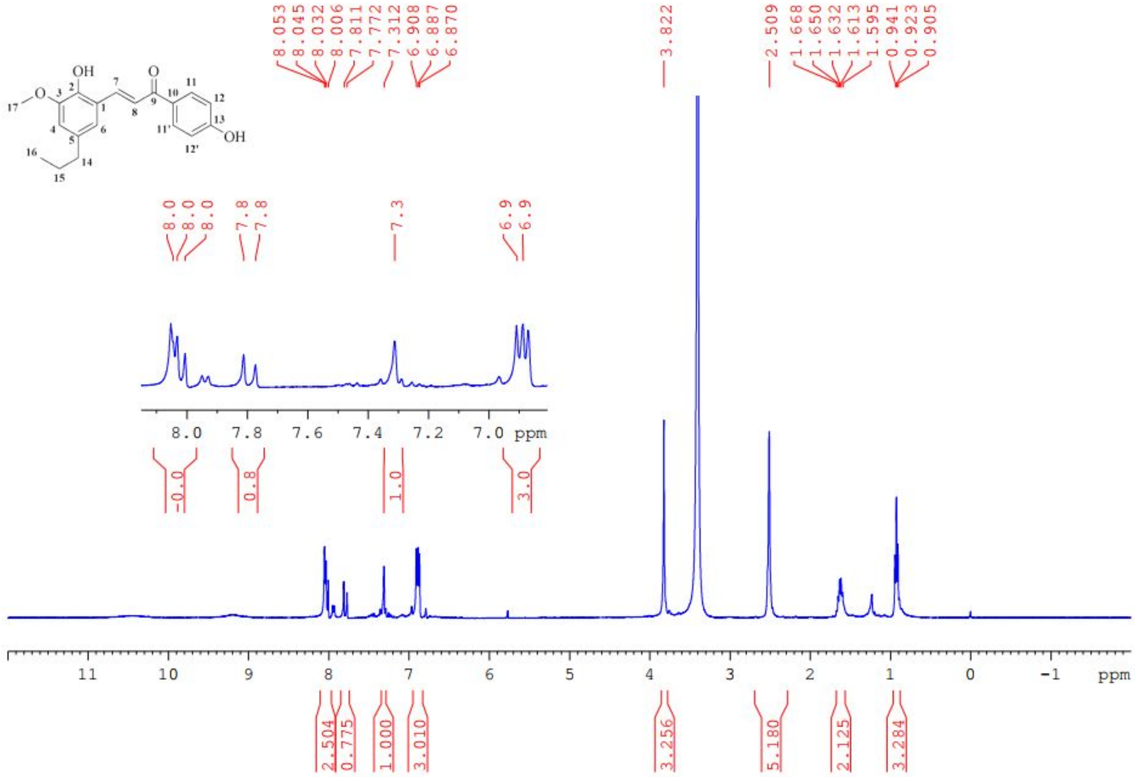

Figure S23 – ^{13}C NMR spectrum of compound 10

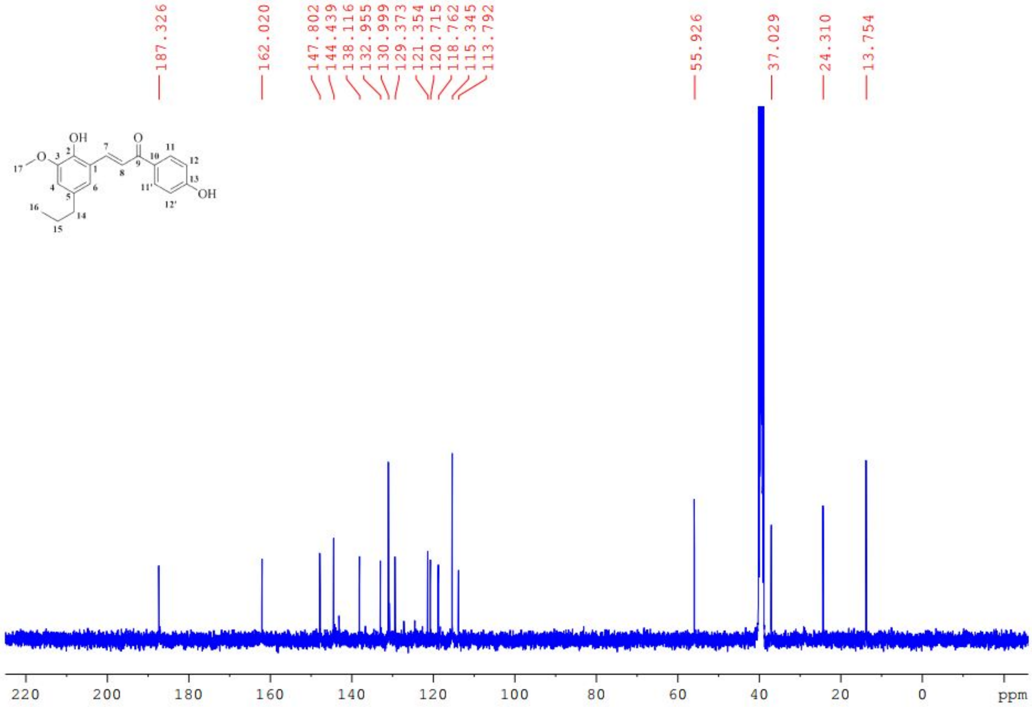

Figure S24 – Infrared spectrum of compound 10

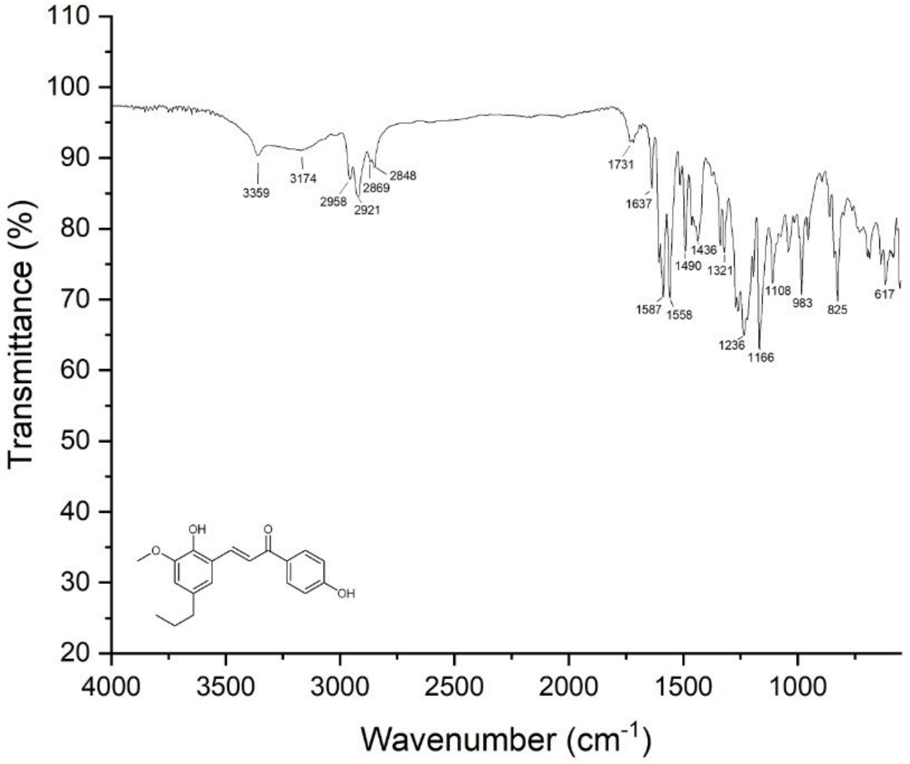

Figure S25 – High resolution mass spectrum of compound 11

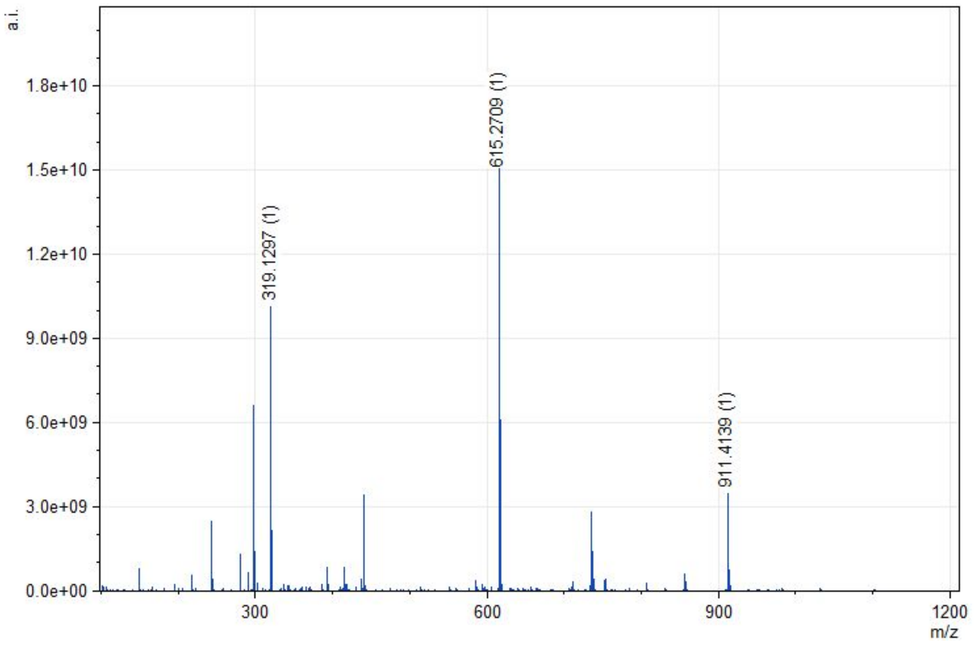

Figure S26 – ^{1}H NMR spectrum of compound 11

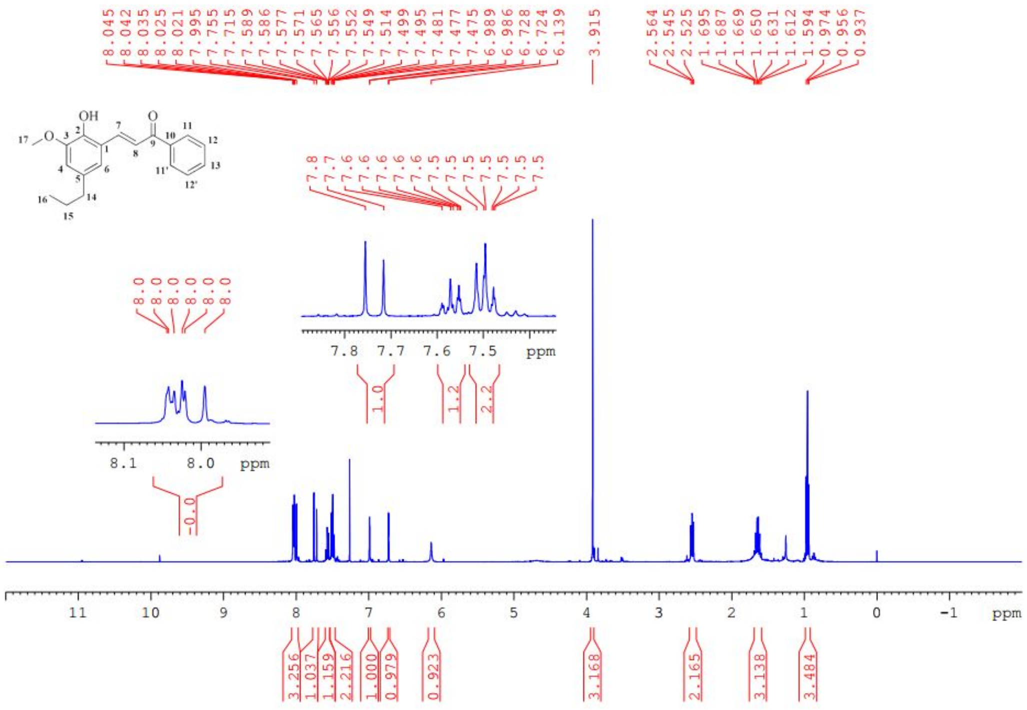

Figure S27 – ^{13}C NMR spectrum of compound 11

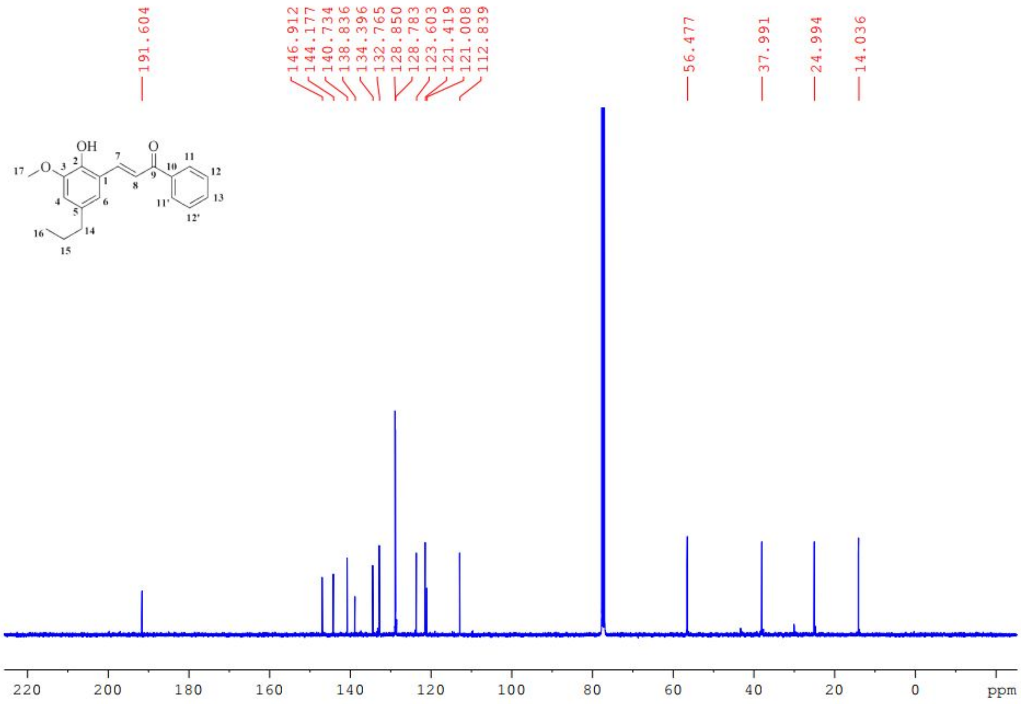

Figure S28 – Infrared spectrum of compound 11

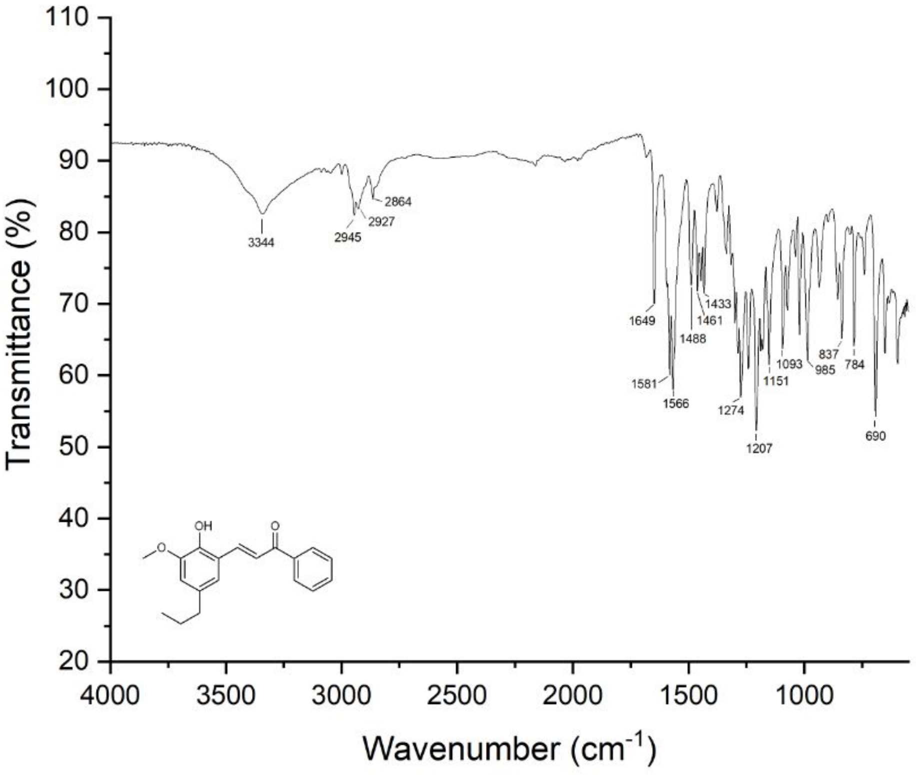

Figure S29 – High resolution mass spectrum of compound 12

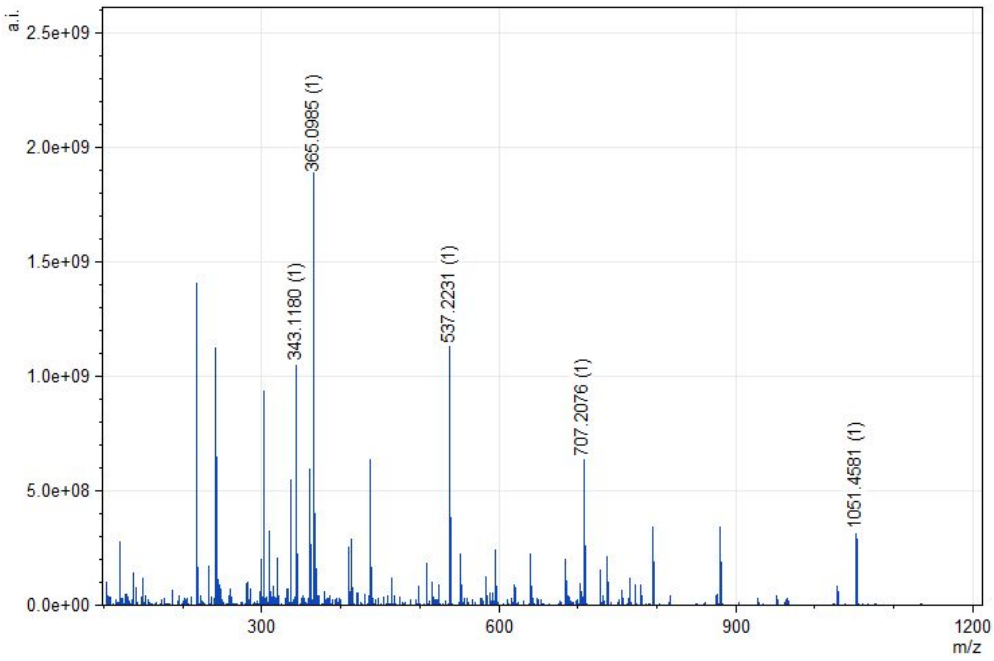

Figure S30 – ^{1}H NMR spectrum of compound 12

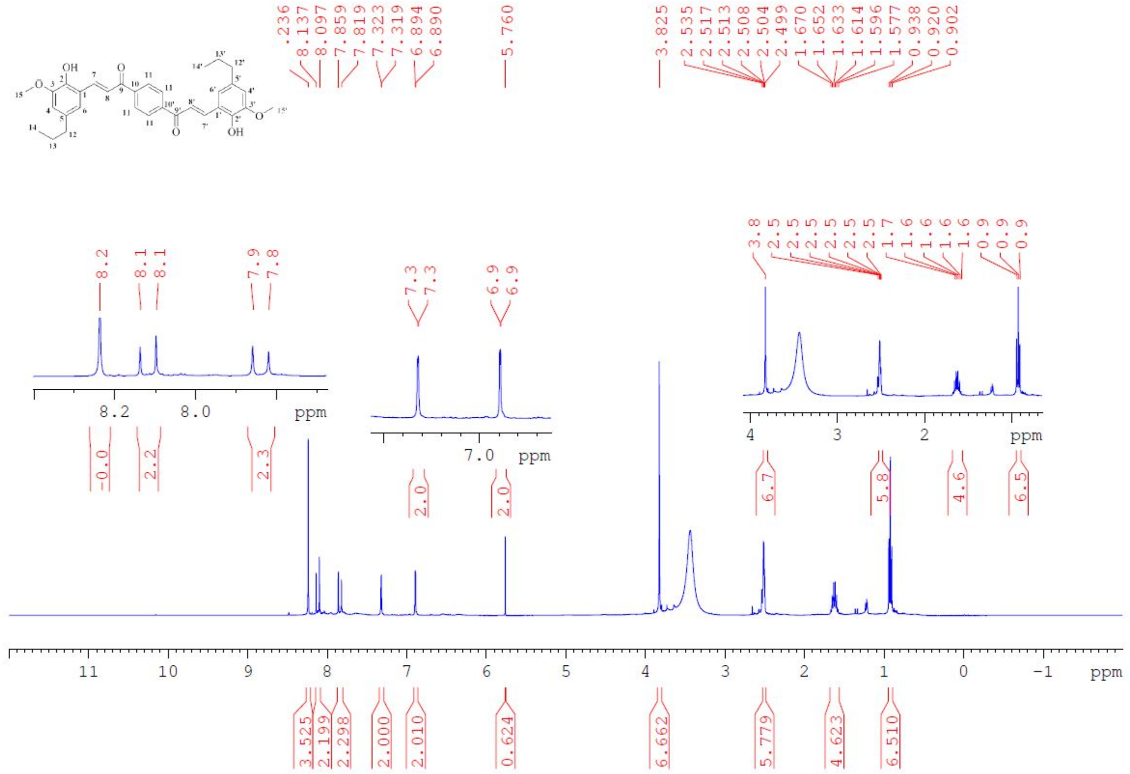

Figure S31 – ^{13}C NMR spectrum of compound 12

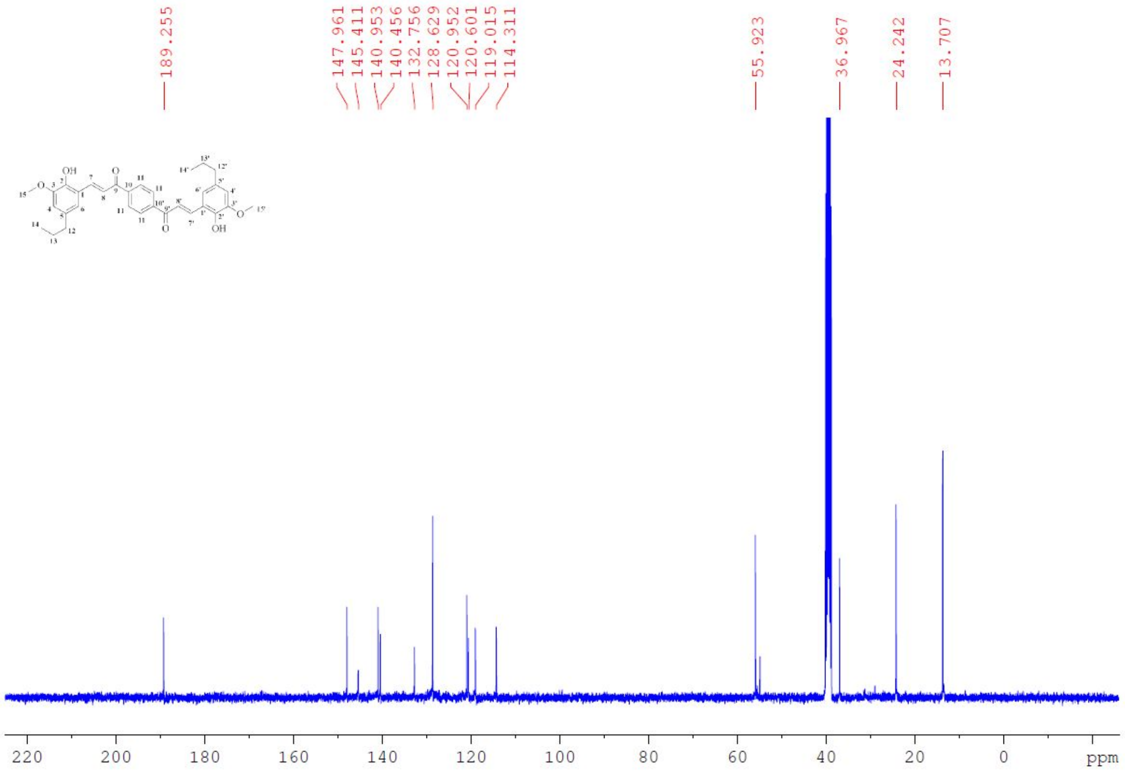

Figure S32 – Infrared spectrum of compound 12

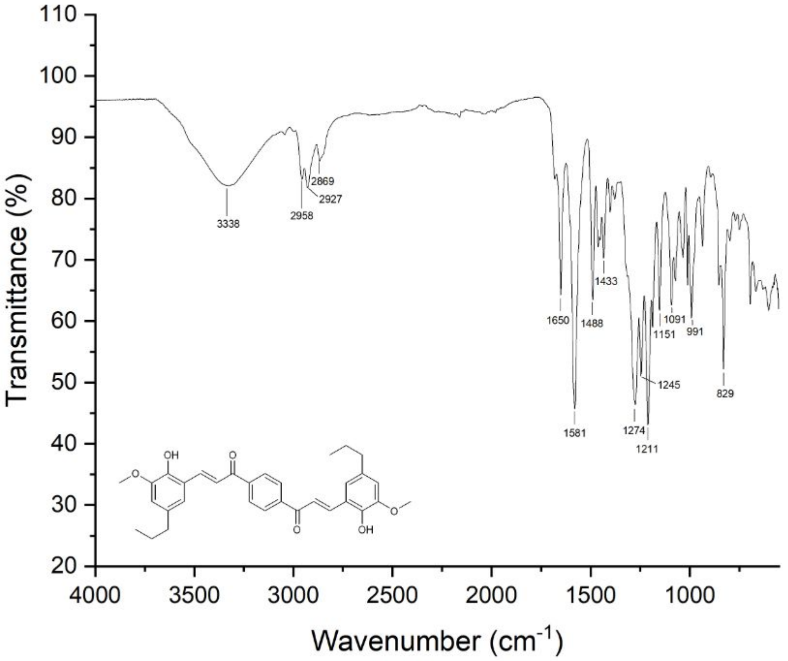

Figure S33 – High resolution mass spectrum of compound 15

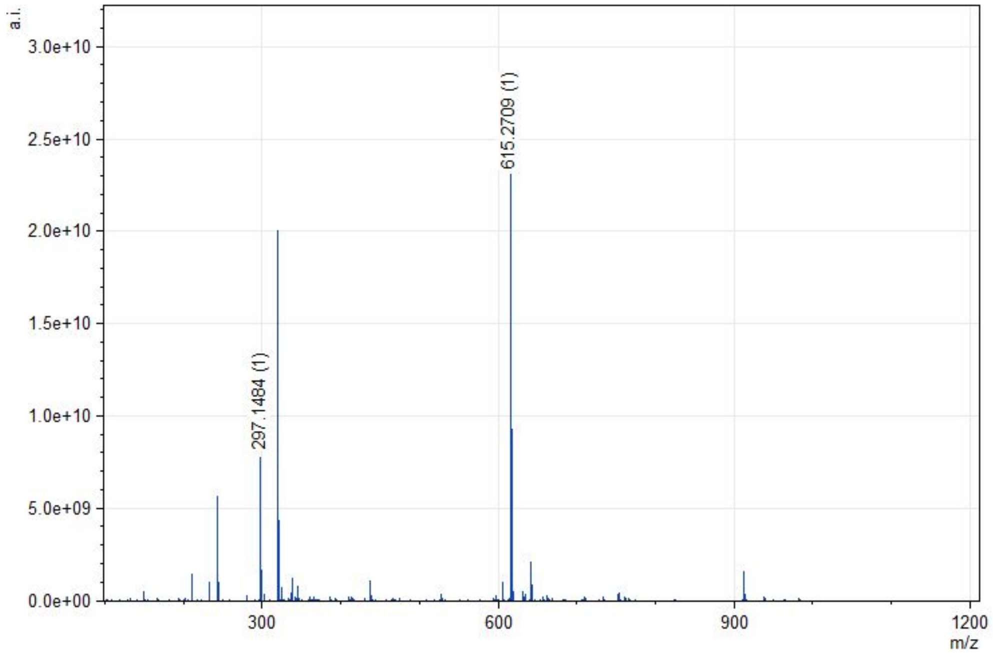

Figure S34 – ^{1}H NMR spectrum of compound 15

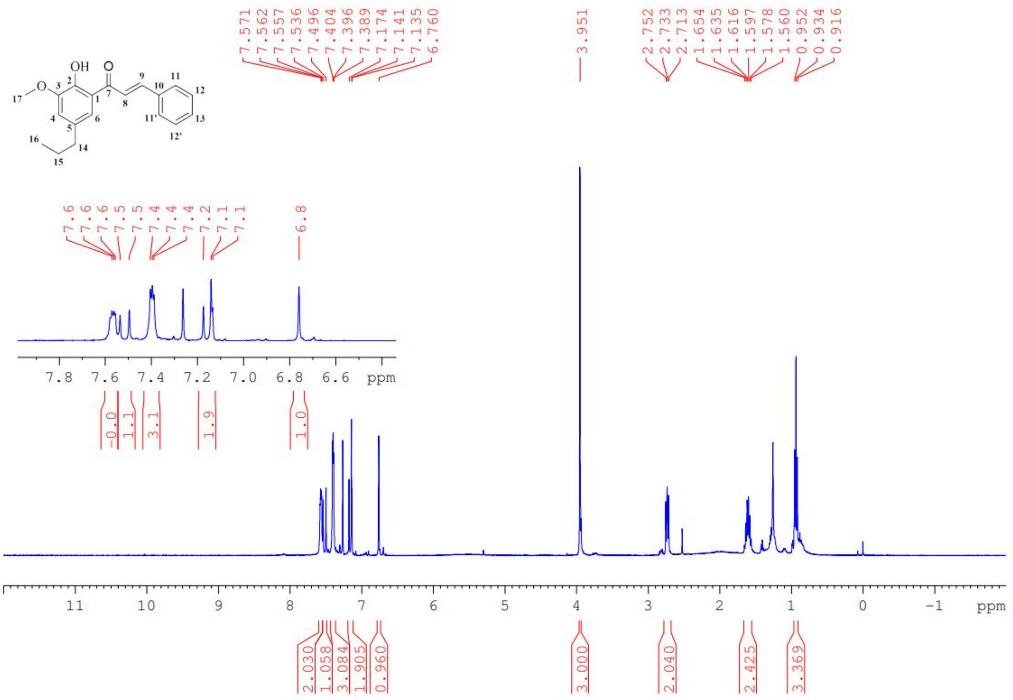

Figure S35 – ^{13}C NMR spectrum of compound 15

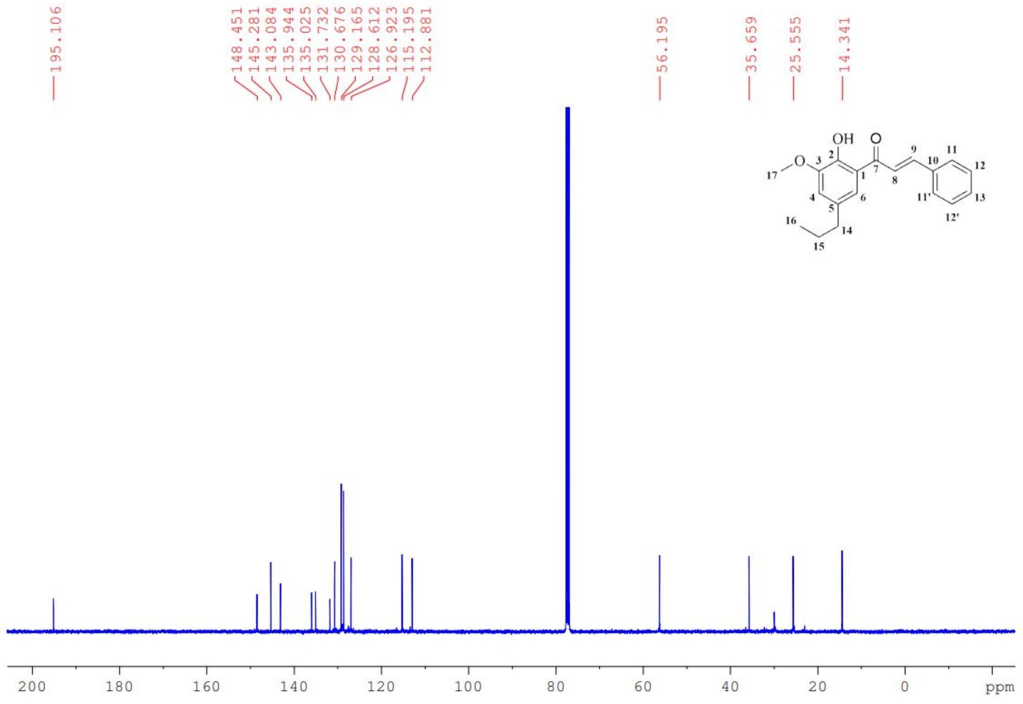

Figure S36 – Infrared spectrum of compound 15

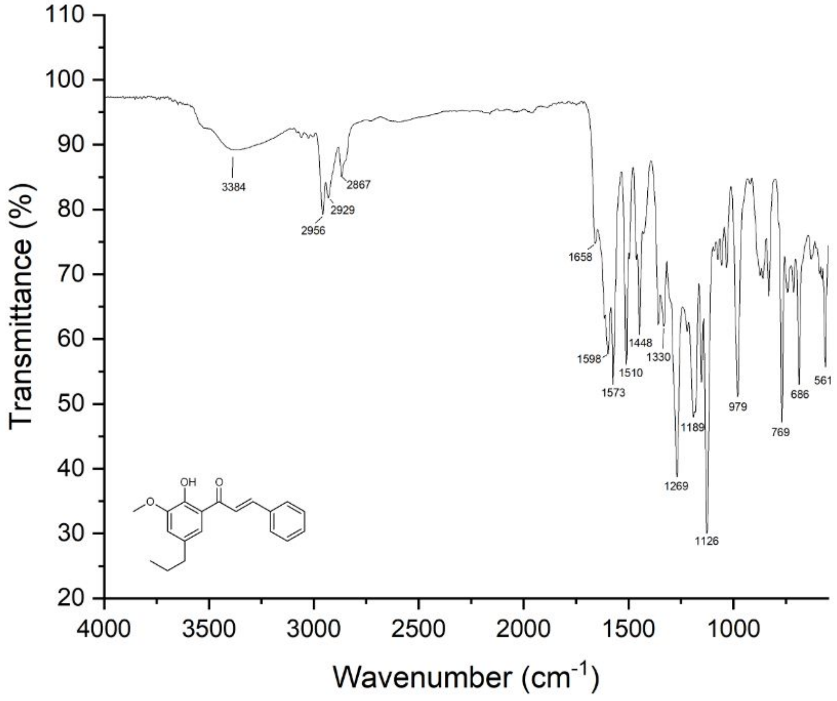

Figure S37 – High resolution mass spectrum of compound 16

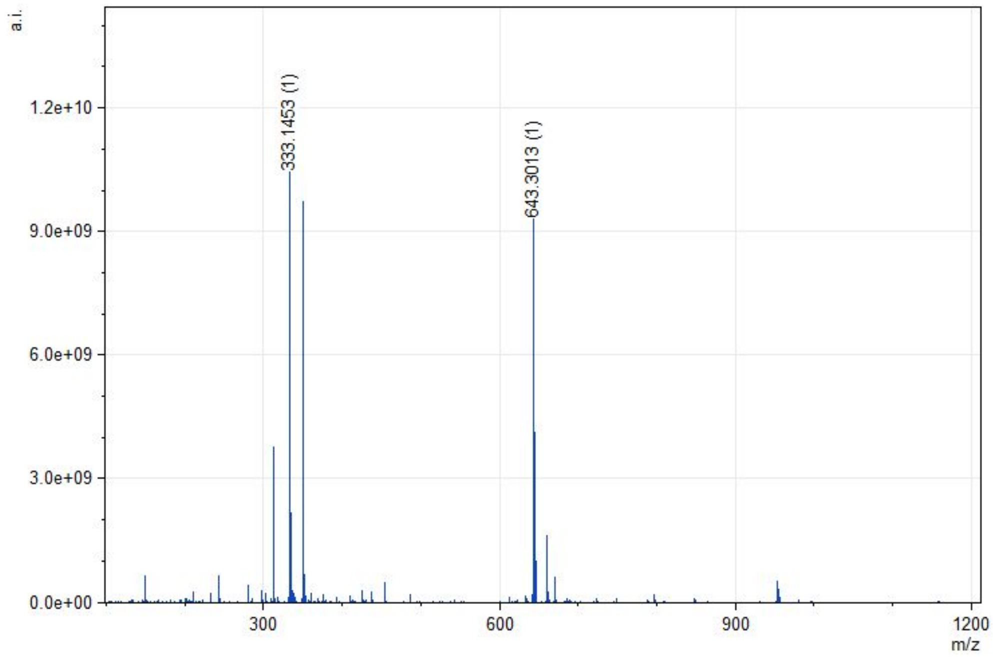

Figure S38 – ^{1}H NMR spectrum of compound 16

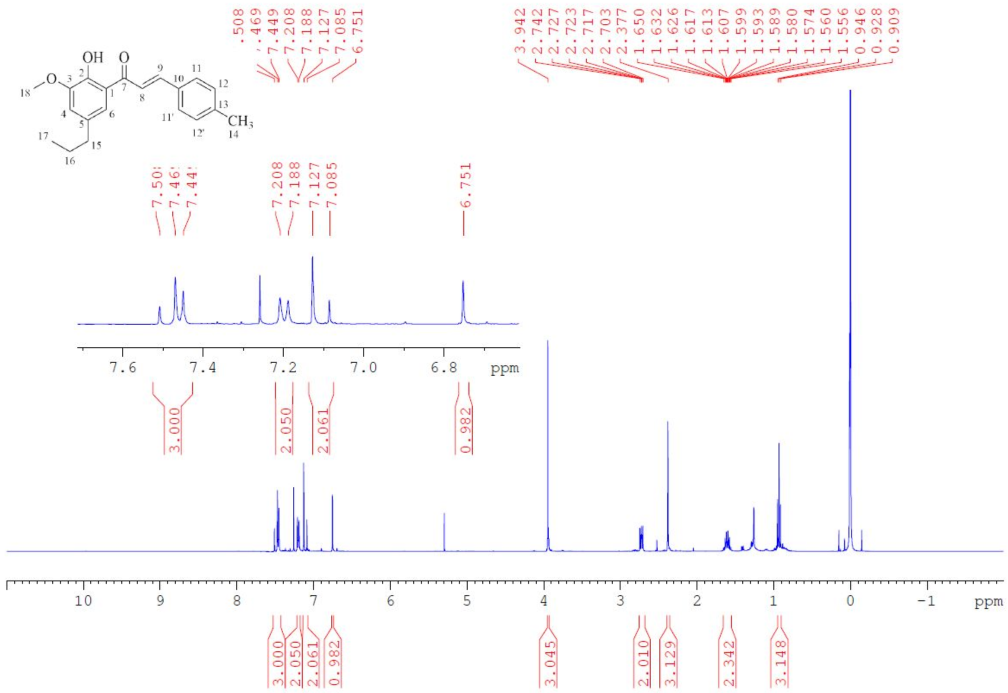

Figure S39 – ^{13}C NMR spectrum of compound 16

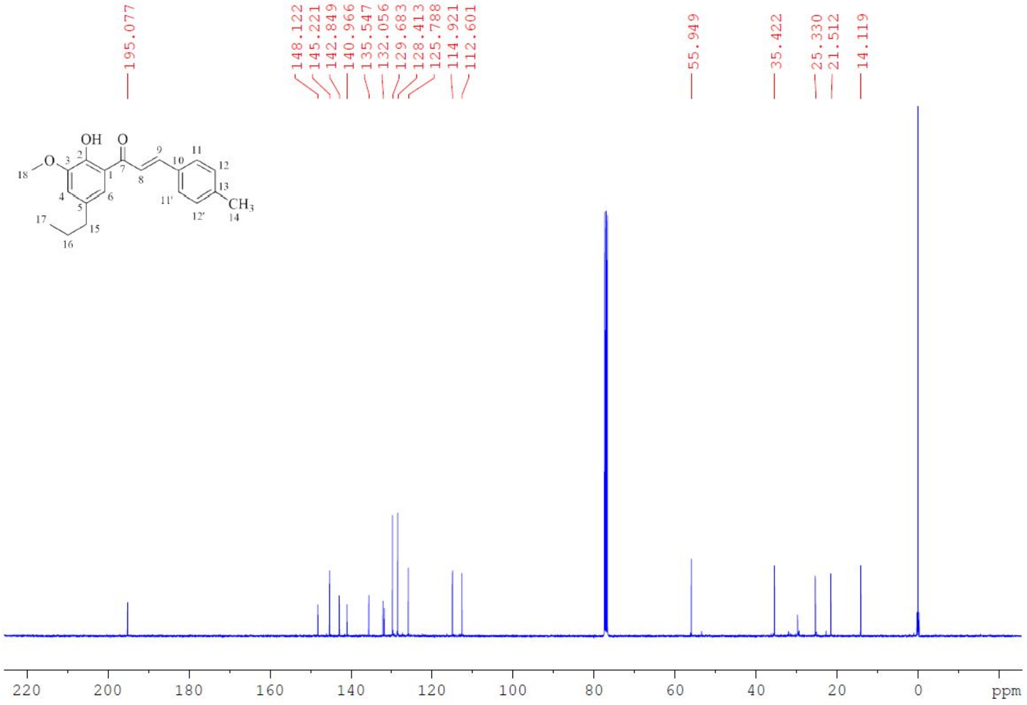

Figure S40 – Infrared spectrum of compound 16

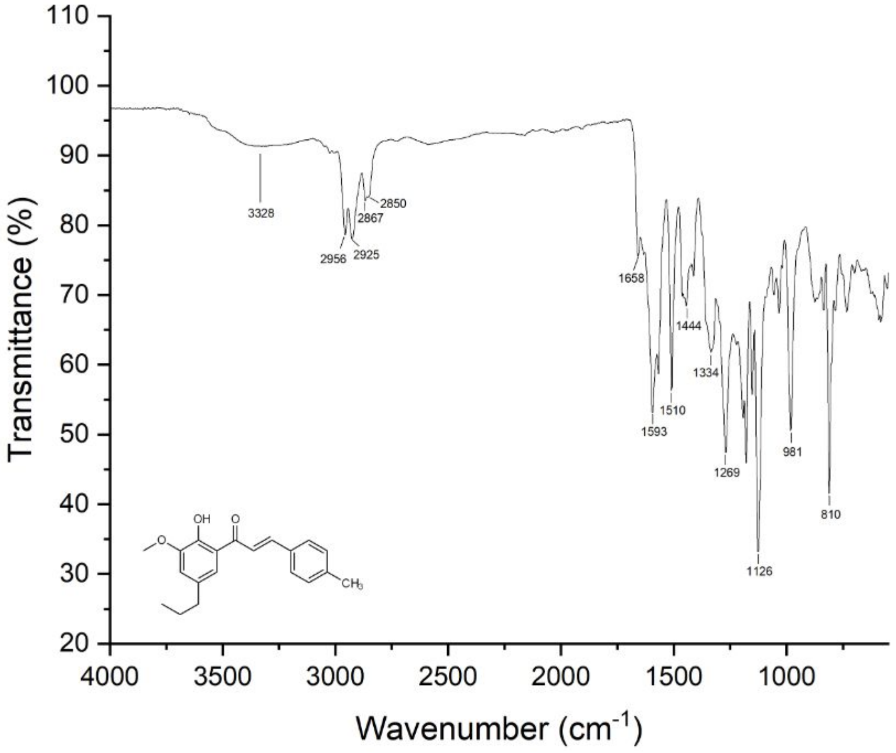

Figure S41 – High resolution mass spectrum of compound 17

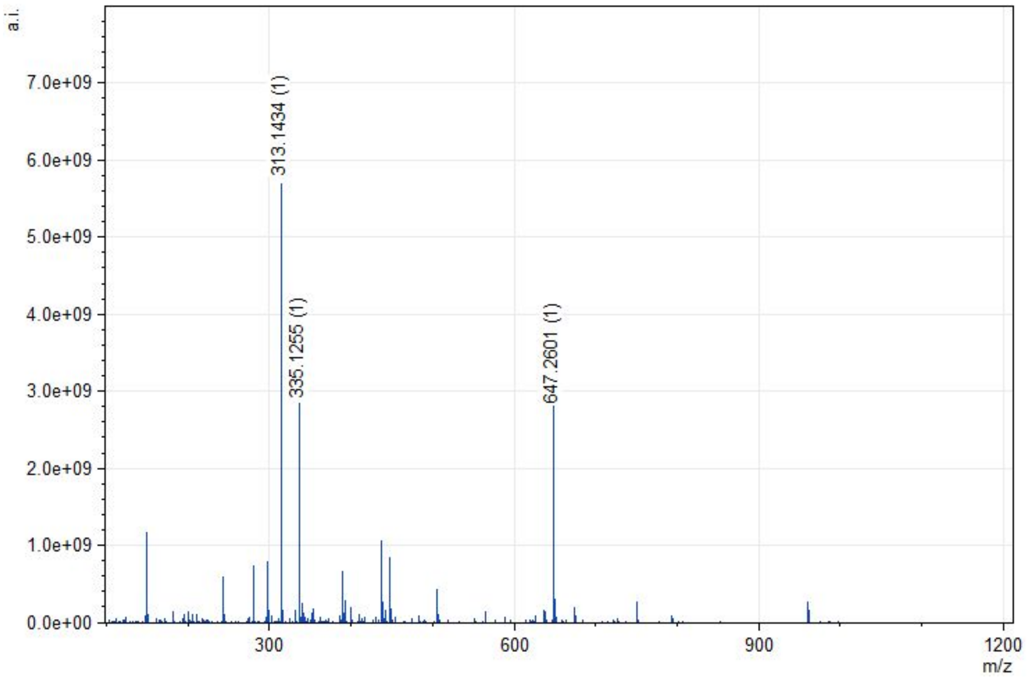

Figure S42 – ^{1}H NMR spectrum of compound 17

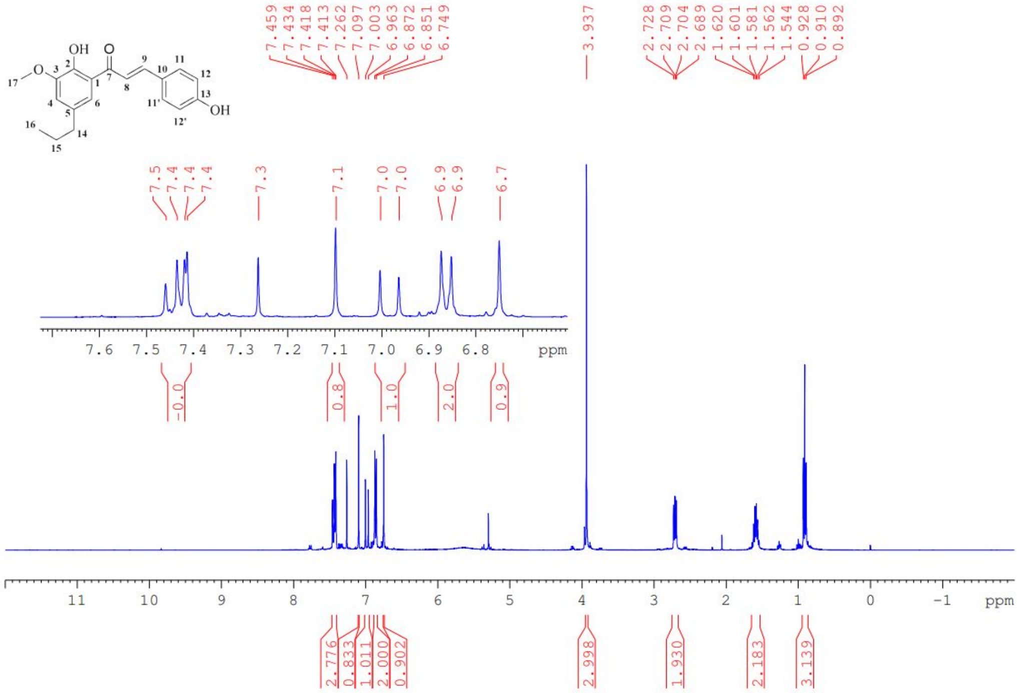

Figure S43 – ^{13}C NMR spectrum of compound 17

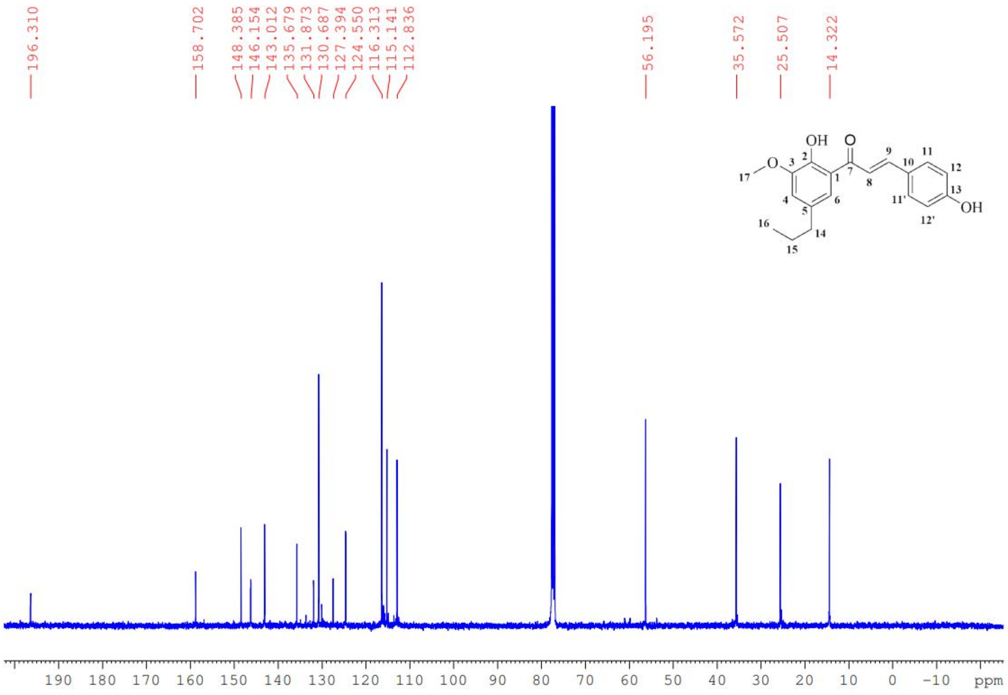

Figure S44 – Infrared spectrum of compound 17

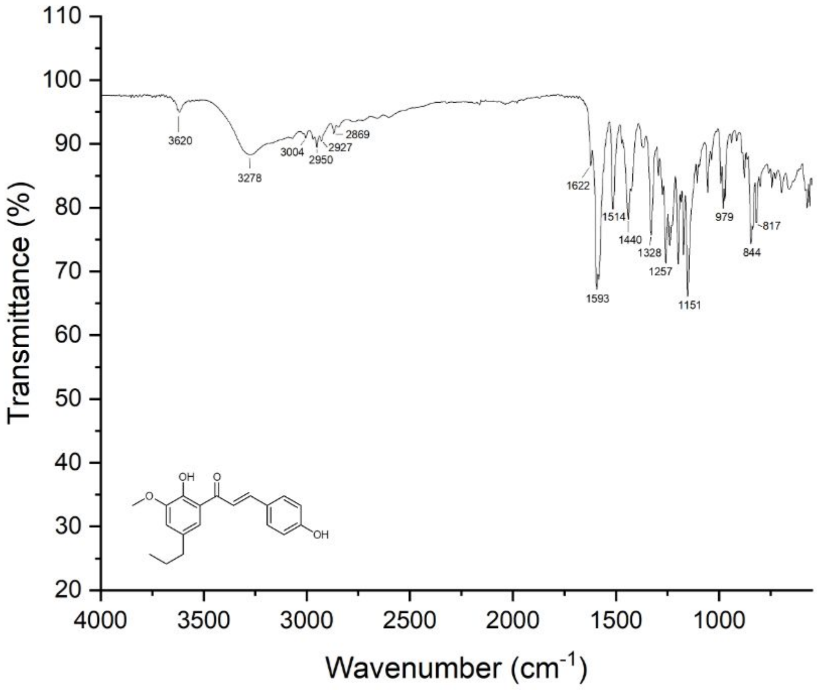

Figure S45 – High resolution mass spectrum of compound 18

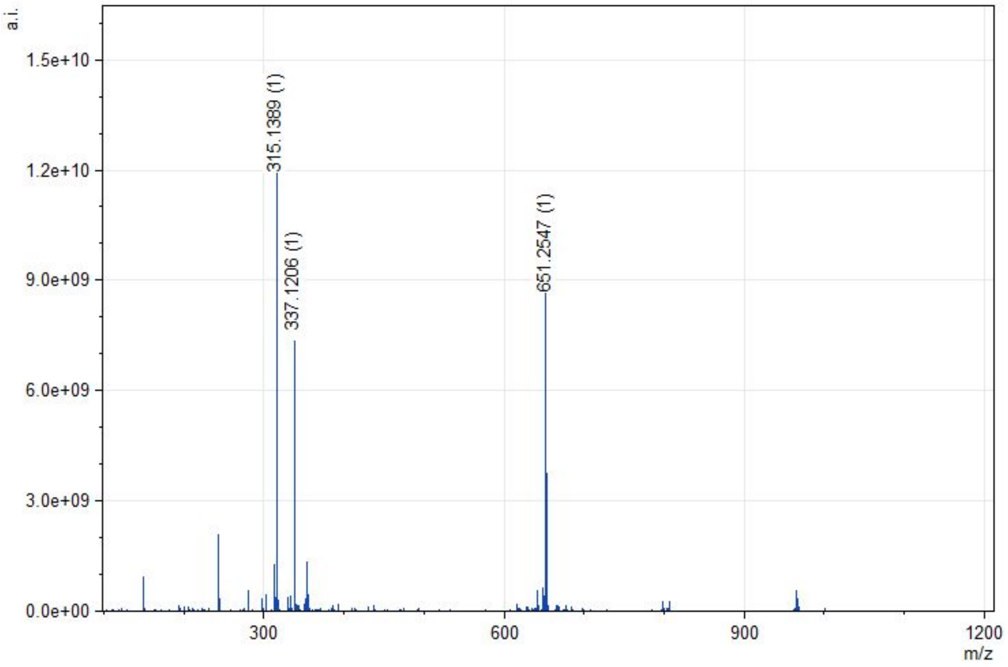

Figure S46 – ^{1}H NMR spectrum of compound 18

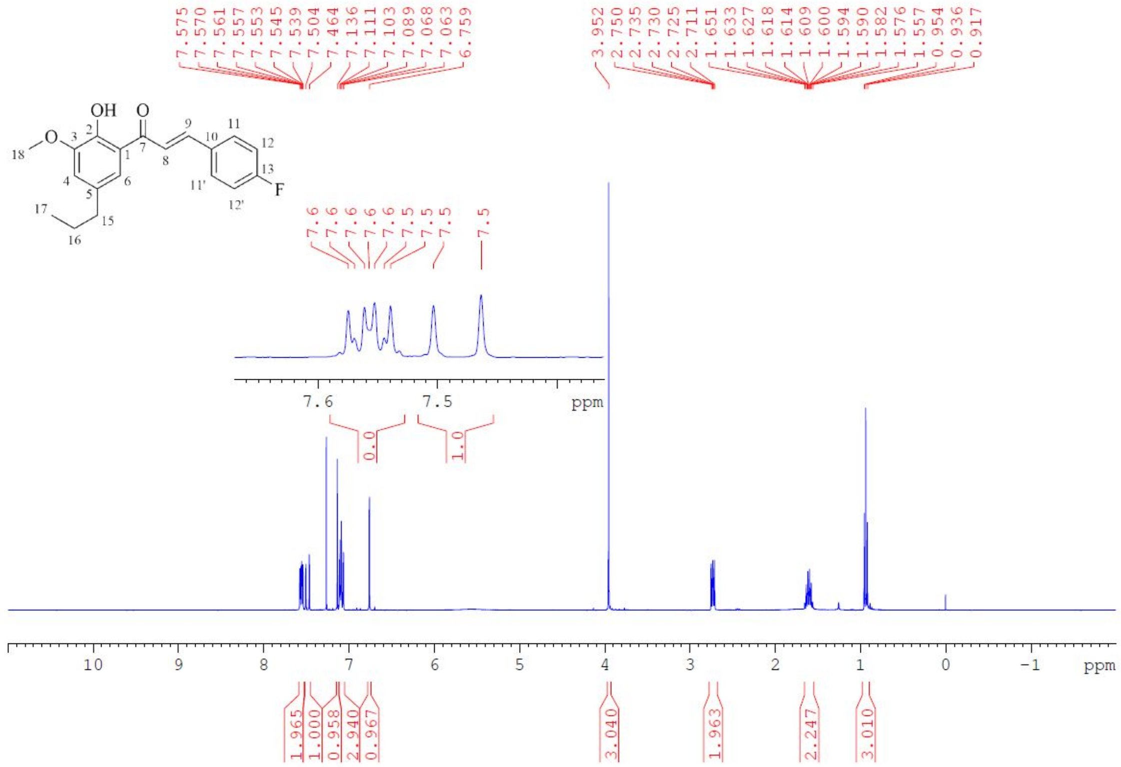

Figure S47 – ^{13}C NMR spectrum of compound 18

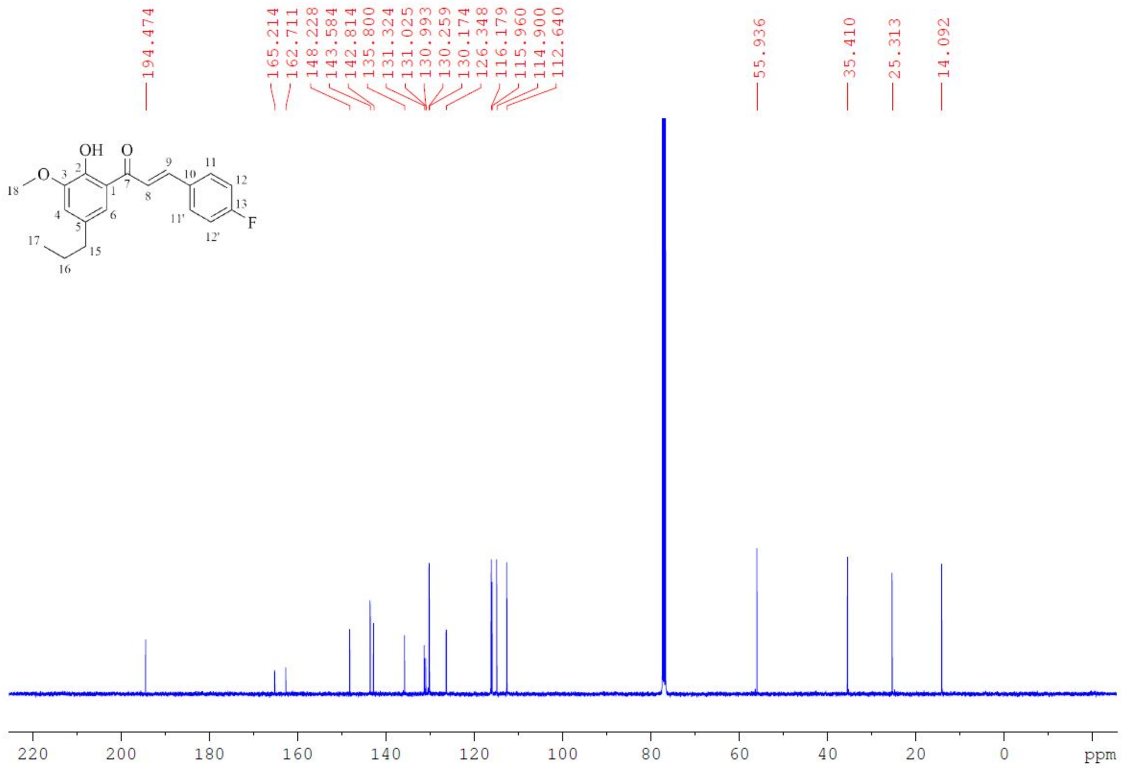

Figure S48 – Infrared spectrum of compound 18

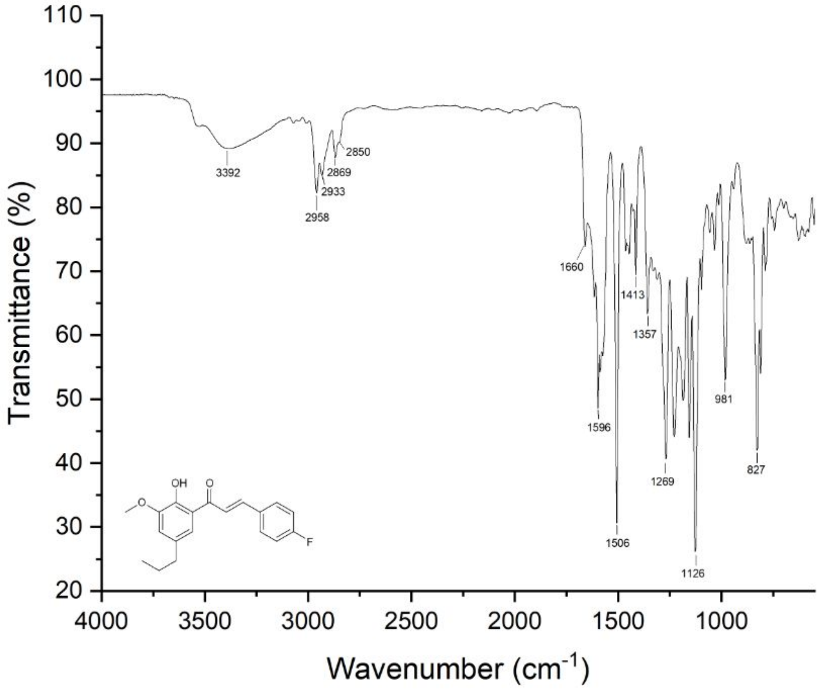

Supplement: Supplementary file 1 [file ao5c08480_si_001.pdf]
